# Asymmetric Doping of a Polyelectrolyte Network Into a Tough Slide‐Ring Hydrogel Membrane to Enhance Sustainable Osmotic Energy Harvesting

**DOI:** 10.1002/smsc.70334

**Published:** 2026-07-09

**Authors:** Subhankar Mandal, Ignacio Lorente Montero, Aseem Milind Visal, Carson J. Bruns

**Affiliations:** ^1^ ATLAS Institute University of Colorado Boulder Boulder Colorado USA; ^2^ Materials Science and Engineering University of Colorado Boulder Boulder Colorado USA; ^3^ Paul M. Rady Department of Mechanical Engineering University of Colorado Boulder Boulder Colorado USA

**Keywords:** blue energy, gradient hydrogel, osmotic energy harvesting, reverse electrodialysis, slide ring gel

## Abstract

Asymmetric ion‐selective membranes are beneficial for harvesting osmotic energy from salinity gradients such as the seawater‐freshwater interface by reverse electrodialysis (RED). Among the various RED membrane technologies, gradient hydrogel membranes can exhibit exceptional performance due to structural features that facilitate ion‐selective transport, but often suffer from poor mechanical properties and/or high internal resistance. Here, a high‐entanglement slide‐ring asymmetric polyelectrolyte double‐network (SRAP‐DN) hydrogel membrane has been prepared by unilateral photopolymerization for osmotic energy harvesting by RED. The membrane can achieve high output power densities >10 Wm^−2^ under the neutral 50‐fold salinity gradient, which can be boosted to >16 Wm^−2^ at pH 12 with a 200‐fold KCl gradient. Unlike most hydrogel membranes, SRAP‐DN is remarkably tough yet extremely high in water content (93%), which lowers internal resistance while benefiting cost and scalability by minimizing polymer content. SRAP‐DN was leveraged to prototype miniature and flexible thin‐film power supplies made of 24 cells connected in series that can harvest osmotic energy from natural seawater and river water to produce >2 V of stable potential. The excellent performance of these tough, water‐rich membranes in blue energy harvesting bodes well for the prospect of low‐cost, eco‐, and bio‐friendly lightweight power supplies for wearable, implantable, or clean energy technologies.

## Graphical abstract

An asymmetric polyelectrolyte hydrogel membrane toughened by an interpenetrating slide‐ring primary network leads to high efficiency in sustainable osmotic blue energy harvesting by reverse electrodialysis.

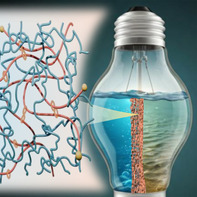

## 1 Introduction

New and reliable materials for renewable energy are continually needed to reduce global dependence on fossil fuels and ensure a sustainable future [1]. “Blue energy” [2, 3], which refers to osmotic energy derived from the chemical potential in aqueous salinity gradients, is considered to be a promising clean energy source due to the abundance of earth’s water resources [4, 5]. Researchers have been investigating various membrane‐based technologies [5, 6] to harvest blue energy at the seawater‐freshwater interface since the seminal work of Pattle in 1954 [7]. Two membrane‐based methods have emerged as sustainable options for salinity gradient power: pressure‐retarded osmosis [8] (PRO) and reverse electrodialysis (RED) [9, 10]. Research on RED systems, which can potentially generate higher power from seawater with less membrane fouling [11], has surged in recent decades [12], while blue energy power plants such as the Afsluitdijk dam in the Netherlands validate [13] RED technology as a viable source of scalable, emission‐free electricity.

Conventional RED energy generators typically utilize a stack of alternating cation‐ and anion‐exchange membranes [14] between two electrodes where electron‐relaying redox reactions occur, driven by the chemical potential associated with ion migration [15]. However, these systems often face challenges of high interfacial resistance and low power density [3, 16], falling short of the targeted 5.0 Wm^−2^ industrial benchmark for the seawater/freshwater salinity gradient [5]. Nanoscale ion‐transport phenomena in biological systems like ion channels [17] and electrocytes [18] have inspired a new generation of nanofluidic RED membranes [19, 21], which can now be constructed from a variety of materials including, for example, polymers [19, 22], silica [20], boron nitride [23, 24], molybdenum disulfide [25, 26], graphene oxide [27], metal‐organic frameworks [28, 29], covalent organic frameworks [30], vermiculite [31], zeolites [32], alumina nanochannels (ANM) [33, 34], polyoxometalates [35], and clay/cellulose [36]. Significant research efforts have revealed how factors such as porosity/pore size [22, 37], surface charge density [23, 33], wettability [38, 39], geometry [40], and other characteristics of nanopores can impact power conversion [41]. Recently, RED energy conversion efficiencies have been enhanced by directionally biased ion transport in nanopores with material asymmetry, such as those of Janus membranes [33, 42, 46], which exhibit diode‐like rectified ionic currents [47, 48]. However, high membrane resistance arising from small pore sizes or insufficient pore coupling at heterogeneous membrane interfaces can adversely affect power density [45, 49], while the complexities of nanofabrication can also limit scale‐up.

Polyelectrolyte hydrogels have emerged as promising membrane materials to enhance osmotic energy harvesting efficiency, owing to high ion selectivities and transport properties attributable to their hydrated charged polymer networks [50, 52]. Despite these benefits, hydrogel membranes often exhibit excessive swelling and inadequate mechanical properties, limiting their viability for long‐term use at scale, which motivates the design of mechanically and chemically durable polyelectrolyte hydrogels. Several generalizable hydrogel strengthening strategies are well known, such as double networks [53, 54], nanocomposite reinforcement [55], physical and covalent dual cross‐linking [56], slide‐ring networks [57], highly entangled networks [58, 59], and combinations thereof [60, 62]. In particular, the double network strategy is utilized to maintain excellent mechanical properties and chemical stability at high water content (>90%) [63, 65]. Although asymmetric hydrogel morphologies are rarely explored to induce an ionic gradient, a few hydrogels with asymmetric membrane compositions exhibiting high energy efficiency and mechanical stability in RED have been reported recently [52, 66, 67].

Building on this promising direction for RED hydrogel membranes, we adapted our recent work [68] on highly entangled slide‐ring double networks with high toughness and water content to create a hydrogel RED membrane with an asymmetric polyelectrolyte gradient in one of the interpenetrating networks. These slide‐ring asymmetric polyelectrolyte double network (SRAP‐DN) hydrogel membranes provide robust mechanical and chemical stability over a wide range of pH and salt concentrations, with high diode‐like ion conductivity and ion selectivity leading to high maximum power densities almost double the commercialization benchmark. A noteworthy feature of the membrane that differentiates it from others in the field is the high water content of 93%; we anticipate that such low polymer content may be beneficial for reducing cost and increasing scalability of RED membranes. Employing these membranes in gelatinized salinity gradients enables the fabrication of a flexible thin‐film wearable power supply that delivers approximately 10 Wm^−2^ of stable power, indicating good candidacy for implantable or wearable electronic devices.

## 2 Results and Discussion

We developed and optimized the synthesis of double network hydrogel membranes comprising a slide‐ring primary network doped asymmetrically with a polyelectrolyte secondary network and then explored the mechanical and osmotic energy‐harvesting properties of these slide‐ring asymmetric polyelectrolyte double network (SRAP‐DN) membranes in a battery of reverse electrodialysis experiments.

### 2.1 Hydrogel Synthesis

**FIGURE 1:**
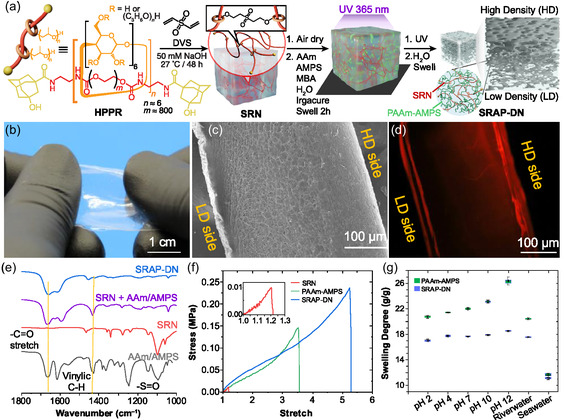
A slide‐ring asymmetric polyelectrolyte double network (SRAP‐DN) membrane. (a) Schematic illustration of synthesis of SRAP‐DN hydrogel by asymmetric photo‐crosslinking of a P(AAm‐AMPS) network interpenetrating a slide‐ring network. (b) Photograph of SRAP‐DN hydrogel membrane showing stretchability and mechanical strength. (c) SEM image of a cross‐sectioned SRAP‐DN hydrogel. (d) Fluorescence microscopic image of a cross‐sectioned SRAP‐DN hydrogel tagged with Rhodamine B dye showing gradient distribution. (e) FTIR spectra of AAm/AMPS monomer mixture, HPPR‐SN, AAm‐AMPS pre‐gel soaked HPPR‐SN network, and final SRAP‐DN hydrogel. (f) Overlaid stress–strain curves of HPPR‐SN and PAAm‐AMPS‐SN single network hydrogels and the SRAP‐DN hydrogel membrane at strain rate of 100 mm min^−1^. (g) Comparison of swelling degree of SRAP‐DN and PAAm‐AMPS‐SN hydrogel in different conditions of pH and aqueous conditions.

A slide‐ring network and a high‐entanglement network were synthesized independently as single networks and as the interpenetrating double network SRAP‐DN (Figure 1).

#### 2.1.1 Synthesis of Single‐Network Hydrogels

The slide‐ring network (SRN) is derived from hydroxypropylated polyrotaxane (HPPR), comprising adamantane‐capped polyethylene glycol (PEG) sparsely threaded by hydroxypropylated *α*‐cyclodextrin (HP*α*CD) rings; the inclusion ratio is estimated to be only 1.6% by ^1^H NMR analysis (Figure S1). When HPPR is dissolved at high polymer content (18–38 wt%) in dilute aqueous NaOH (50 mM), the HP*α*CD rings can be cross‐linked by divinylsulfone to form a tough and stretchable SRN hydrogel. The SRN hydrogel is employed as the host for a second entangled network of photo‐polymerized acrylamide (AAm) and acrylamidopropane sulfonic acid (AMPS) in the double‐network strategy. As a basis for comparison, single‐network PAAm‐AMPS hydrogels were prepared in the absence of a host SRN network by photo‐polymerization of highly concentrated aqueous AAm and AMPS solutions with low concentrations of methylenebisacrylamide (MBA) cross‐linker and Irgacure 2959 photoinitiator. These conditions are known to result in long polymer chains with relatively sparse cross‐links, resulting in physical entanglements that outnumber covalent cross‐links [58]. Much like slide‐ring networks, these highly entangled networks exhibit high toughness and extensibility (vide infra) attributable to a slip‐link mechanism at chain entanglement sites [58], resembling that of ring sliding [69].

#### 2.1.2 Synthesis of Slide‐Ring Asymmetric Polyelectrolyte Double Network (SRAP‐DN) Hydrogel

Slide‐ring asymmetric polyelectrolyte double network (SRAP‐DN) hydrogel membranes were synthesized (Figure 1a) by photo‐polymerization. The air‐dried SRN network was swollen in an aqueous pre‐gel solution of highly concentrated AAm (15–26 M) and AMPS (1–2 M) monomers with a low concentration of MBA cross‐linker (0.0005–0.002 mol%) and Irgacure 2959 photoinitiator (0.0005 mol%), then cured by a directionally biased UV light source to create a density gradient in the second network (Figure 1a). Under unilateral illumination with a 365‐nm UV‐LED light on the pregel‐swollen SRN atop a glass slide covered in black tape, the intensity of light within the sample gradually decreases from the surface to the base of the hydrogel. Finally, the cured sample was equilibrated in a large excess of water for 24 h with two changes of the water bath to wash out unreacted monomers to obtain the swollen SRAP‐DN hydrogel. FTIR spectra (Figure 1e) show that the vinylic peak at 1425 cm^−1^, seen in the AAm/AMPS‐soaked pre‐gel, disappears as expected after photo‐polymerization generates the entangled PAAm‐AMPS second network of the SRAP‐DN hydrogel. After the hydrogel membrane is washed and allowed to fully swell in water, it remains mechanically robust (Figures 1b and S2) despite its high water content of 93 wt%.

#### 2.1.3 Morphology of SRAP‐DN

Anisotropy in the form of a structural gradient can be advantageous for the cation transport properties of hydrogel membranes [52, 66, 67]. As a result of the pre‐gel’s internal UV illumination gradient introduced during unilateral photopolymerization, a higher density of the PAAm‐AMPS polymer is expected on the side of the membrane nearest to the UV light source. This density gradient of the second network was observed in cross‐sectioned samples of the SRAP‐DN hydrogel membrane by scanning electron microscopy (SEM) images (Figure 1c). It revealed larger and more numerous cracks and pores on the low‐density (LD) side of the membrane relative to the high‐density (HD) side (Figure S3). To further visualize the gradient formation and charge distribution in the cross section, the SRAP‐DN hydrogel was soaked in a solution of red‐fluorescent Rhodamine B dye; fluorescence microscopic images revealed a fluorescence gradient (Figure 1d) attributable to greater association between positively‐charged Rhodamine B and negatively‐charged sulfonate groups in the PAAm‐AMPS matrix on the HD side of the membrane. This Rhodamine B gradient thus reflects a change in the density of the polyelectrolyte network, which would be expected to correspond to a gradient in space charge envisioned to support ionic rectification and ion transport [52].

### 2.2 Mechanical Properties of the Hydrogel Membranes

Tough membranes with high water content may be desirable for RED applications in order to minimize material costs and maximize ionic conductivity, yet most hydrogels become weak and brittle at high water contents exceeding 90 wt%. The SRAP‐DN was investigated in this context because the maintenance of high toughness, recoverability, and extensibility at high water content is a key feature of high‐entanglement slide‐ring double network hydrogels [68].

#### 2.2.1 Mechanical Properties of the SRN Single Network

SRN hydrogels are known to exhibit remarkable stretchability and toughness [57], which is attributed to the pulley effect [70], whereby the sliding motions of cross‐linked rings non‐destructively dissipate the energy of mechanical stress in the network. Indeed, the as‐synthesized SRN hydrogels exhibit a good range of stretchability and tensile stress at DVS cross‐linker concentrations of 1.5–4.0 wt% (Figure S4a) and high HPPR content of 18%–38% (Figure S4b) in the pre‐gel solution. The optimal SRN samples (Figure S4c), constituting 38 wt% HPPR and 2.5 wt% DVS, displayed high ultimate tensile strength (*σ*_max_) of 0.25 MPa and extensibility (*λ* = 12). However, as‐synthesized SRN hydrogel membranes, which have low water content of 60 wt%, swell to beyond 90 wt% water content at equilibrium and become weak and brittle (see insets in Figures 1f and S4c), which motivated our recent work on slide‐ring double‐network hydrogels that leverage chain entanglement to sustain high loads at high water content [68].

#### 2.2.2 Mechanical Properties of the High‐Entanglement PAAm‐AMPS Single Network

The mechanical properties of the single‐network PAAm‐AMPS polyelectrolyte hydrogels were optimized (Figure S5) by varying the concentration of monomer at 1:0.06 AAm:AMPS mol ratio (15–26 M, Figure S5a,b), MBA cross‐linker (0.0005–0.002 mol%, Figure S5c,d), and Irgacure 2959 photoinitiator (0.00025–0.001 mol%, Figure S5e,f) in the pre‐gel solution. The optimized PAAm‐AMPS single‐network hydrogel, which was obtained with high monomer concentration (26 M), 0.001 mol% MBA and 0.0005 mol% Irgacure initiator, exhibits high tensile strength (*σ*_max_ = 0.16 MPa) and extensibility (*λ* = 3.55) for a hydrogel swollen to a very high water content of 94 wt% at equilibrium, consistent with the toughening expected in networks where entanglements outnumber cross‐links [58].

#### 2.2.3 Mechanical Properties of SRAP‐DN Hydrogels

Different hydrogel formulations were investigated to synthesize an optimized SRAP‐DN sample with the total monomer concentration fixed at ∼26 M and photoinitiator concentration fixed at 0.0005 mol%, while varying [MBA] and [AMPS] in the pre‐gel swelling solution (Figure S6). The ultimate tensile stress increases from approximately 0.14 to 0.22 MPa as [MBA] increases from 0.0005 to 0.002 mol% (Figure S6a). The maximum elongation, ranging from 4.02 to 5.15, was maximized at 0.001 mol% MBA (Figure S6b). At 0.001 mol% MBA, [AMPS] was increased from 2.5 to 7.5 mol% (relative to total monomer, fixed at 26 M), revealing that the mechanical properties begin to deteriorate extensively beyond 5.5 mol% AMPS (Figure S6c). Under the optimized synthetic conditions ([MBA] 0.001 mol%, [AMPS] 5.5 mol%), the swollen SRAP‐DN hydrogel exhibits high tensile strength of 0.23 ± 0.01 MPa and 5.15 ± 0.13 ‐fold extensibility (Figure 1f), as well as adequate stiffness of 85 ± 5 kPa, high toughness of 462 ± 9 kJm^−3^, and high fracture energy of 765 ± 28 Jm^−2^.

#### 2.2.4 Synergistic Effect of Double Networking

A comparison (Figure S7) of the mechanical properties of each network reveals that the mechanical toughness of the SRAP‐DN hydrogel arises from a synergy between the two entangled slip‐link networks, since the toughness (Figure S7b), stiffness (Figure S7c), and fracture energy (Figure S7d) of SRAP‐DN are all much greater than the sum of the two component networks. The toughness of SRN (0.95 kJm^−3^) and PAAm‐AMPS (155 kJm^−3^) sum to 156 kJm^−3^, only 33% of the 462 kJm^−3^ toughness of SRAP‐DN (Figure S7b). The stiffness of SRN (0.6 kPa) and PAAm‐AMPS (45 kPa) sum to 45.6 kPa, only 53% the 85 kPa stiffness of SRAP‐DN (Figure S7c). The fracture energies of SRN (0.2 Jm^−2^) and PAAm‐AMPS (255 Jm^−2^) sum to 255.2 Jm^−2^, only 33% the 765 Jm^−2^ fracture energy of SRAP‐DN (Figure S7d). This synergy is consistent with our prior findings in high‐entanglement slide‐ring double network hydrogels [68]. All three swollen networks exhibit similarly high water content; the 93.2% water content of SRAP‐DN is an average of the SRN (92.5% water) and PAAm‐AMPS (94.2% water) single networks (Figure S7a). Therefore, the remarkable enhancement in mechanical properties observed in SRAP‐DN compared with the sum of its component networks cannot be attributed to differences in water content.

#### 2.2.5 Mechanism of Synergistic Toughening

The mechanism underlying the synergistic toughening of the high‐entanglement slide‐ring double network has been investigated in detail in a companion study on the parent HESRDN hydrogel [68], which combines experimental controls with fiber bundle and Rubinstein–Panyukov modeling to elucidate the dual slip‐link mechanism. In brief, two distinct physical slip‐link motifs contribute to the mechanical synergy: (i) The pulley effect [57] of mobile cyclodextrin ring cross‐links in the slide‐ring primary network, which redistribute tension along the polymer backbone rather than concentrating stress at fixed junctions, and (ii) the entangled chain slip‐links [58] of the sparsely cross‐linked acrylic‐based secondary network, which delocalize stress through chain reptation. The slide‐ring primary network acts as a *reversible* sacrificial network—unlike conventional double networks in which the sacrificial primary network dissipates energy through irreversible bond rupture, the mobile ring cross‐links can slide to accommodate local stress concentrations without sustaining permanent damage. This reversible sacrificial mechanism, combined with the entanglement‐mediated stress delocalization of the secondary network, produces toughness, stiffness, and fracture energy far exceeding the sum of the component networks, as demonstrated quantitatively through comparison with fixed‐cross‐link control double networks [68]. The mechanically robust SRAP‐DN hydrogel also shows a much lower swelling degree than the PAAm‐AMPS single network in seawater, river water, and fresh water across a wide pH range of 2–12 (Figure 1g). The maintenance of excellent mechanical properties at very high water content across such a broad range of aqueous conditions for the SRAP‐DN hydrogel indicates potentially good candidacy for RED membrane applications.

### 2.3 Osmotic Energy Conversion Performance of SRAP‐DN Hydrogel Membrane

**FIGURE 2:**
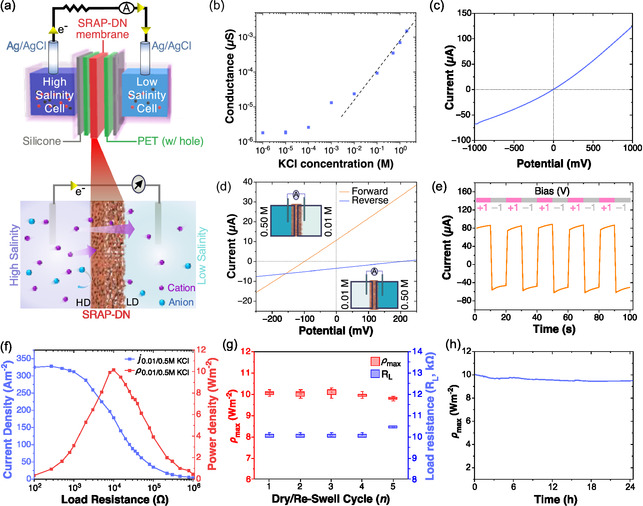
Transmembrane ionic properties of SRAP‐DN. (a) Schematics of the SRAP‐DN membrane/solution/electrode assembly to study osmotic energy, as well as cation selectivity and ion transport through the SRAP‐DN membrane separating high and low salinity electrolyte. (b) Ionic conductance as function of KCl concentration. The dashed line represents the bulk property of the electrolyte. (c) *I–V* curve of SRAP‐DN membrane with symmetric 0.50 M KCl on both sides (no Nernstian electrode contribution to the EMF), depicting a rectification factor of ≈2. (d) *I–V* curves of SRAP‐DN membrane in 0.50/0.01 M KCl gradients, with the HD side of the membrane facing the 0.50 M solution (forward) or the 0.01 M solution (reverse). Cation transport numbers calculated by subtracting the Nernstian Ag/AgCl contribution (Section S2) are *t*_+_ ≈0.54 (forward) and *t*_+_ ≈1.0 (reverse). (e) Current–time curve of the SRAP‐DN based RED setup with 0.01/0.50 M KCl electrolyte depicting cyclic stability of the transmembrane ion transport property. External biases of +1 and −1 V were alternately applied for 10 s for consecutive five cycles. (f) Current density and power density plots of SRAP‐DN hydrogel‐based RED device in the 50‐fold (0.50 M/0.01 M) KCl salinity gradient, measured in the forward orientation; Section S2 provides the breakdown of membrane and electrode contributions to the cell EMF. (g) Variation in maximum power density and load resistance of a SRAP‐DN hydrogel‐based RED device while using the same SRAP‐DN hydrogel over multiple drying and re‐swelling cycles. (h) Stability of the SRAP‐DN RED system over a 24‐h period in the 0.5 M/0.01 M KCl salinity gradient at 10 kΩ load resistance.

**FIGURE 3:**
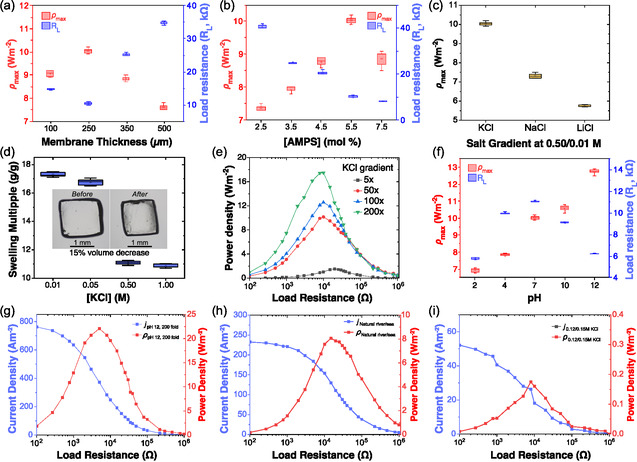
Effects of varying parameters on osmotic energy harvesting performance of the SRAP‐DN hydrogel membrane based RED setup. (a) Maximum output power density and load resistance of different SRAP‐DN hydrogel membranes with varying thickness of the optimized hydrogel membrane having 5.5 mol% AMPS across 0.01 M versus 0.50 M KCl salinity gradient. (b) Maximum output power density and load resistance with varying AMPS molar ratio in the hydrogel (thickness 250 μm) in the 0.01 M/0.50 M KCl salinity gradient. (c) The effect of different electrolytes in energy harvesting performance of the optimized SRAP‐DN hydrogel membrane using the (0.5/0.01 M) salinity gradient. (d) The swelling multiple of SRAP‐DN with increasing [KCl] shows the anti‐swelling property of the membrane in saline conditions. Inset photographs showcase the 15% volume decrease of SRAP‐DN after being transferred from fresh water into 1.0 M KCl. (e) Power density plots of the RED system with increasing KCl gradient up to 200‐fold against 0.01 M KCl. (f) Variation of maximum power density and load resistance of the optimal SRAP‐DN hydrogel membrane while varying pH from 2 to 12 in the 0.5/0.01 M KCl gradient. (g) Current density and power density plots of the SRAP‐DN RED system at pH 12 in the 200× KCl gradient. (h) Current density and power density plots of the SRAP‐DN RED system in a gradient of natural sea water and river water. (i) Current density and power density plots of the SRAP‐DN RED system in a physiologically‐relevant low salinity gradient with 0.15 M/0.12 M NaCl electrolytes.

We fabricated a 3D‐printed electrochemical apparatus (Figure S8a,b) with two compartments separated by a membrane assembly comprising a SRAP‐DN hydrogel film sandwiched between PET and silicone rubber sheets. The PET sheets in contact with the hydrogel each have a microscopic hole (Figure S8c) approximately 200 μm in diameter, while the outer silicone and 3D‐printed components have macroscopic holes, to enable transmembrane diffusion between the cells. This apparatus was used to conduct transmembrane ionic transport and osmotic energy harvesting measurements (Figures 2 and 3).

#### 2.3.1 Ion Conductance

The high water content of the SRAP‐DN membrane is envisioned to facilitate ionic conductance, while the negatively charged PAAm‐AMPS polymer network is expected to facilitate cation selectivity (Figure 2a). The ionic conductance of the SRAP‐DN hydrogel increased by three orders of magnitude, from 2 × 10^−6^ to 2 × 10^−3^ μS, as the KCl concentration increased from 1 μM to 1 M. This transmembrane ionic conductance is known to play a crucial role in osmotic energy conversion [52]. In the plot of conductance versus [KCl] (Figure 2b), the ionic conductance starts to deviate from the bulk value (indicated by the dashed line) as [KCl] falls below 0.1 M. The dashed line, representing a linear fit of conductance at higher concentrations, illustrates the bulk behavior of ionic conductance, which is proportional to the ionic concentration of the electrolyte. However, under hyposaline conditions (less than 0.1 M), the high surface‐to‐volume ratio of micro/nano‐porous channels allows surface charges to dominate ionic conductance, resulting in significant enhancements that appear to be independent of the bulk ionic concentration [45, 71]. Such unique behavior of conductance suggests that the transmembrane ionic transport process is governed by the charged groups present on the membrane surfaces [66, 72, 73].

#### 2.3.2 Ionic Current Rectification

The asymmetric density gradient of the negatively charged PAAm‐AMPS polymer network in SRAP‐DN is expected to facilitate directional cation‐selective transport (Figure 2a), rectifying ionic current from the high‐density (HD) to the low‐density (LD) side of the membrane. The K^+^‐selective property of the SRAP‐DN membrane is apparent in linear sweep voltammetry (LSV) with 0.5 M KCl in both compartments (Figure 2c), since the K^+^ current at positive bias is ∼2x that of the Cl^−^ current at negative bias, corresponding to a rectification factor [37] of 2. When the SRAP‐DN membrane separates two compartments of different salinity, the K^+^ ions, which are attracted to the  − SO_3_^−^ group of the AMPS monomers, pass through the membrane from high to low concentration, thereby generating osmotic current (Figure 2a). The asymmetric gradient of charge and porosity in the membrane offers a preferential direction of K^+^ transport via possible formation of ionic diode [33]. To prove this directionally biased ion transport, we performed LSV experiments with the membrane separating 0.5 M and 0.01 M KCl solutions in both directions (Figure 2d). With the HD side exposed to the high‐salinity 0.5 M KCl solution (forward direction), the open‐circuit voltage (*V*_oc_) and the short‐circuit current (*I*_SC_) measured 100.2 mV and 9.8 μA, respectively. With the HD side facing low‐salinity 0.01 M KCl solution (reverse direction), *I*_SC_ decreased to 4.9 μA (Figure 2d), while the *V*_oc_ increased to 185 mV. The internal resistance of the membrane in the forward and reverse directions, determined by Ohm’s law, were 10.1 kΩ and 37.7 kΩ, respectively. Subtracting the activity‐corrected Nernstian contribution of the Ag/AgCl electrodes in the chloride gradient (*V*_elec_ ≈ 92 mV, computed using tabulated mean ionic activity coefficients [74]; see Supporting Information) from the measured *V*_oc_ values yields the membrane‐attributable EMF in each orientation, from which the cation transport number *t*_+_ can be calculated using $V_{\text{mem}}=(2t_{+}-1)(RT/F)\ln\;(a_{\text{high}}/a_{\text{low}})$. The transport number is the standard electrode‐independent measure of intrinsic membrane cation selectivity. In the forward orientation, *V*_mem_ ≈ 8 mV gives *t*_+_ ≈ 0.54, indicating weak cation selectivity in the orientation where the membrane provides the lowest internal resistance. In the reverse orientation, *V*_mem_ ≈ 93 mV gives *t*_+_ ≈ 1.0, demonstrating that the SRAP‐DN membrane approaches the ideal‐selectivity limit when the HD side faces the dilute compartment. The pronounced asymmetry in *t*_+_ between the two orientations corroborates the ionic rectification observed in Figure 2c, which itself is independent of electrode contributions because it is measured with symmetric 0.5 M KCl on both sides of the membrane. The repeatability of current values in both +1 and −1 V bias over five consecutive cycles (Figure 2e) confirms the stable ion transport property through the membrane [75].

#### 2.3.3 Osmotic Energy Conversion Measurements

The harvested energy conversion efficiency of the SRAP‐DN hydrogel membrane was assessed by employing 0.5 M KCl and 0.01 M KCl as the high‐ and low‐salinity solutions, simulating artificial seawater and river water. The electrical performance was measured through a circuit consisting of an external load, RED device, and electrochemical station in series (Figures 2a and S8). The maximum output power density (*ρ*_max_) and current density (*j*) are calculated by (*I* ^2^ *R*_L_/*S*) and *I*/*S*, respectively, where *I* represents the measured current, *S* denotes the testing area of the membrane, and *R*_L_ is external load resistance. The *ρ*_max_, *j* and *R*_L_ were measured reaching 10.1 Wm^−2^, 325 Am^−2^, and 10 kΩ (Figure 2f) which is comparable and somewhat higher than the reported state‐of‐the‐art nanofluidic membranes measured under same conditions (Table S1) [22, 26, 33, 34, 37, 44, 45, 51, 52, 66, 76, 82]. The reported power density includes the Nernstian contribution of the Ag/AgCl electrodes to the cell EMF, as is standard for hydrogel‐based RED measurements in chloride electrolytes (Tables S1 and S2); a quantitative breakdown of the electrode and membrane contributions is provided in Supporting Information Section S2. We note that the effective membrane test area of *S* ≈ 3 × 10^4^ μm^2^ (corresponding to a ∼200 μm diameter pore) is small relative to practical membrane areas, and power densities measured through such micro‐apertures may be enhanced relative to larger‐area membranes due to reduced concentration polarization and edge effects [49]. This aperture size is the standard test configuration in the nanofluidic and hydrogel RED literature, and the hydrogel‐membrane studies summarized in Tables S1 and S2 use comparable effective test areas; reporting the present 10.1 Wm^−2^ value at the same aperture size therefore preserves comparison with prior work, while leaving the absolute deployment‐scale performance open. Direct characterization of the area‐dependence of the SRAP‐DN power density, by measurement across a range of effective membrane areas spanning the micro‐ to macro‐aperture regimes, is an important direction for future scale‐up studies.

#### 2.3.4 Membrane Stability in RED

To evaluate the stability of the membrane against drying and prolonged use, we evaluated the performance over multiple cycles of drying and re‐swelling (Figure 2g) and 24 h of continuous operation (Figure 2h). The SRAP‐DN hydrogel membrane exhibited minimal changes in *ρ*_max_ and load resistance over five consecutive cycles of air‐drying and re‐swelling (Figure 2g). The system also showed high long‐term stability (Figure 2h), with the power output decreasing by only 6.4% to 9.45 Wm^−2^ over a 24 h period of continuous operation. Together, these stress tests suggest that the high toughness and elasticity of the SRAP‐DN network render it suitable for sustained energy harvesting despite the very low polymer content (7%) of the membrane.

#### 2.3.5 Effect of Membrane Thickness

The effect of membrane thickness on energy harvesting efficiency was assessed (Figures 3a and S9a) by varying the membrane thickness from 100 to 500 μm in the 50‐fold KCl salinity gradient. The optimum thickness of the hydrogel was ∼250 μm, beyond which the output power density decreases with thickness due to a rise in transmembrane ionic resistance (Figure 3a). The lower efficiency observed in the case of the thinner 100 μm membrane can be attributed to an inadequate charge gradient as evident from the increase of internal resistance.

#### 2.3.6 Effect of AMPS Concentration

The concentration of AMPS in the second network of SRAP‐DN influences the osmotic energy harvesting performance of the system significantly, since the charged sulfonate sidegroups of this monomer provide the space charge gradient. Thus, increasing [AMPS] from 2.5 to 5.5 mol% (with respect to the total monomer concentration in the pre‐gel soaking solution) leads to a rise in *ρ*_max_ (Figures 3b and S9b), resulting in a peak value of 10.1 Wm^−2^, nearly double the commercial benchmark of 5 Wm^−2^. Simultaneously, the internal load resistance declines, reaching a minimum of 10 kΩ at 5.5 mol% AMPS (Figure 3b). Beyond 5.5 mol% AMPS, the SRAP‐DN membranes became more weak and fragile; therefore the observed decline in *ρ*_max_ at 7.5 mol% AMPS is likely caused by network inhomogeneities or microscopic membrane damage. These observations highlight the trade‐off when increasing AMPS content in the membrane; an optimal AMPS content will provide sufficient negative charge to enhance cation‐selective ion current without embrittling the network beyond its service capabilities.

#### 2.3.7 Effect of the Cation

Figures 3c and S9c show that the energy conversion characteristics of the membranes depend on the cation of the electrolyte, where the maximum power output in a 50‐fold salinity gradient is highest for KCl (10.1 Wm^−2^), falling to 7.25 Wm^−2^ for NaCl and 5.72 Wm^−2^ for LiCl. The difference in the output power density for different electrolytes can be attributed to their diffusion coefficients, which follow the order: K^+^ (1.96 × 10^−9^ m^2^s^−1^) > Na^+^ (1.33 × 10^−9^ m^2^s^−1^) > Li^+^ (1.03 × 10^−9^ m^2^s^−1^) [83]. In common with other cation‐selective membranes, faster cation diffusion leads to more effective charge separation, thus giving KCl an advantage with regard to high power density [45, 52].

#### 2.3.8 Effect of Salinity Gradient

We evaluated the performance of the SRAP‐DN hydrogel membrane with varying salinity gradients. As expected, the SRAP‐DN membrane exhibits an anti‐swelling effect (Figure 3d) against KCl because charge screening causes the polyelectrolyte network to shrink. This deswelling at elevated KCl concentrations also increases the strength and stiffness of the SRAP‐DN hydrogel in saline conditions (Figure S10). These anti‐swelling properties are well‐suited for blue energy harvesting, demonstrating that the membrane will resist damage in both low‐ and high‐salinity conditions. Figure 3e shows how the power density that can be extracted by the SRAP‐DN‐based RED system increases in greater KCl concentration gradients. As expected, both power density and current density increase along with the concentration gradient, attributed to the enhanced driving force for osmotic ion transport. The maximum value of both current density and power density was achieved at 200‐fold (2.00/0.01 M) KCl gradient, reaching 780 Am^−2^ and 22.5 Wm^−2^. These power densities significantly surpass the commercial benchmark of 5 Wm^−2^, suggesting that the SRAP‐DN hydrogel can efficiently capture blue energy across a broad range of salinity gradients available from different salt‐lake or seawater gradients around the world.

#### 2.3.9 Effect of pH

Using the standard 0.5/0.01 M KCl concentration gradient, we investigated the impact of varying pH on the output power density of SRAP‐DN hydrogel‐based RED system (Figures 3f and S9d). The maximum power output increases substantially with pH, reaching an ultrahigh value of *ρ*_max_ = 12.8 Wm^−2^ at pH 12. This phenomenon can be attributed to the conversion of  −SO_3_H (belonging to the AMPS monomer segments) into  −SO_3_^−^ by adding base, which ionizes the network pores, thus increasing K^+^ ion transport and therefore power. By contrast, in acidic conditions (pH 2) the  −SO_3_^−^ moieties are mostly protonated (the pKa of AMPS is 2) [84] which hinders K^+^ ion transport through the membrane. This effect is further evidenced by the reduction of load resistance at higher pH values (Figure 3f), pointing to increased ionic conductivity in alkaline conditions. While the pH 12 conditions yield impressive power output, we note that maintaining such alkaline conditions would pose practical challenges for long‐term deployment; the neutral‐pH performance of 10.1 Wm^−2^ in the 50‐fold KCl gradient is therefore more representative of realistic operating conditions.

#### 2.3.10 Comparison With Other RED Membranes

The performance of the optimized SRAP‐DN membrane in the 50‐fold KCl gradient, representing the seawater‐river water interface, is compared with that of other all‐hydrogel, nonhydrogel, and heterogeneous membranes [22, 26, 33, 34, 37, 44, 45, 51, 52, 66, 67, 77, 82, 85] in the same salinity gradient in Figure S11 and Tables S1 and S2. The SRAP‐DN RED system exhibits a maximum power output (10.1 Wm^−2^) that is among the highest values yet reported, while the load resistance of 10 kΩ is among the lowest values yet reported. The SRAP‐DN membrane is not only among the most efficient performers in osmotic energy harvesting, but also the lowest in polymer content (7%) and highest in water content (93%) among the hydrogel‐based examples (see Table S2 and Figure S11b,c), which presumably helps lower the internal resistance in ionic current by providing ample hydrated space for ions to migrate. Despite this very high water content, which typically would lead to prohibitively weak hydrogels without the built‐in slip‐link mechanisms [68], SRAP‐DN exceeds the competing RED hydrogel membranes in toughness and stretchability (Figure S11d,e). Simultaneously, achieving low polymer content in hydrogel membranes such as SRAP‐DN will support the minimization of cost and maximization of fabrication scalability, since the polymer is far more expensive and scarce than the water.

### 2.4 SRAP‐DN Membranes in Osmotic Energy Conversion Applications

**FIGURE 4:**
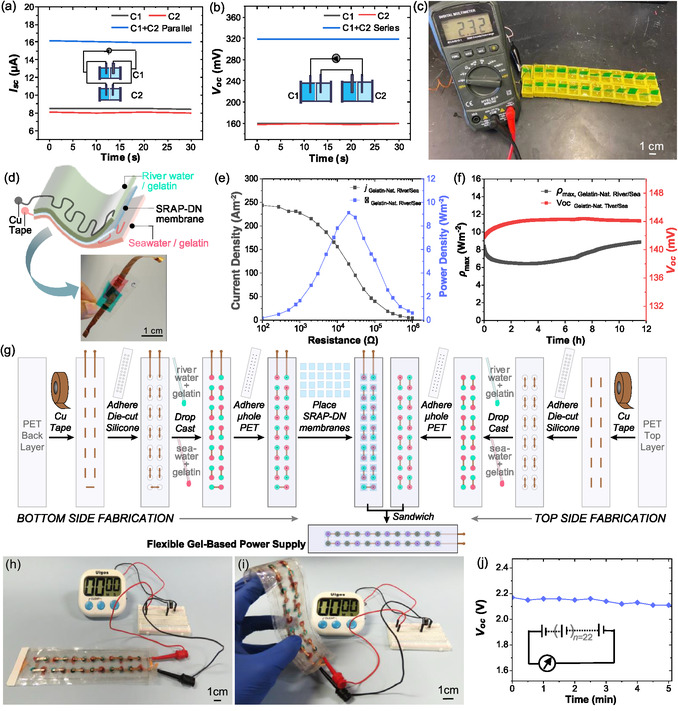
Demonstrations of SRAP‐DN‐based RED devices for energy harvesting in the natural seawater/river water salinity gradient. (a) Short circuit current of two centimeter‐scale RED cells consisting of natural seawater and river water connected in parallel. (b) Open‐circuit voltage of two centimeter‐scale RED cells consisting of natural sea and river water connected in series. (c) Photograph of 24 centimeter‐scale RED cells connected in series to a digital multimeter, which shows a voltage output of 2.32 V. (d) Schematic of a thin‐film gelatin‐based RED device with the SRAP‐DN membrane separating gelled electrolyte solutions. (e) Current density and power density plots of the gelatin‐based SRAP‐DN RED device with Cu tape electrodes at pH 12; Section S2 of the Supporting Information discusses how these measurements relate to the Ag/AgCl‐based intrinsic membrane performance reported in Figures 2 and 3. (f) Stable power output and open‐circuit voltage of the gelatin‐based RED device over a 12 h period of operation. (g) Schematic of a fabrication process to generate thin‐film power supplies from arrays of gelatinized natural seawater and river water droplets sandwiched with an array of small SRAP‐DN membranes. (h) Photograph of a 24‐cell wearable thin‐film SRAP‐DN RED power supply based on the natural gelatinized seawater/river water gradient powering the LCD screen of a digital clock. (i) Photograph of the same thin‐film device continuing to power the digital clock upon deformation by hand. (j) *V*_oc_ plot showing stable power for the wearable device over a 5‐min period of operation.

To demonstrate feasibility and proof‐of‐concept applications of the SRAP‐DN membrane in blue energy harvesting, we evaluated osmotic energy harvesting performance in a number of real‐world conditions (Figure 3h–i) and prototyped some practical power supplies (Figure 4), including flexible gel‐based thin‐film devices, by connecting SRAP‐DN‐based RED cells together in parallel and in series.

#### 2.4.1 Seawater/River Water Salinity Gradient

The SRAP‐DN hydrogel’s applicability in natural conditions was evaluated using natural seawater and river water. Under these conditions, the *ρ*_max_ was 8.2 Wm^−2^ with a maximum current density of 230 Am^−2^ (Figure 3h), which is 15% lower than the artificial 50‐fold gradient but still well above the commercially acceptable benchmark. The lower power output in the natural gradient can be attributed to the difference in ion composition between natural seawater, which contains high levels of Na^+^. Importantly, the SRAP‐DN hydrogel retains its high long‐term stability in these unpurified natural waters, demonstrating excellent stability and endurance over a 24 h period of operation (Figure S12).

#### 2.4.2 Physiological Conditions

The high efficiency, high water content, and good biocompatibility of the polymer components of the SRAP‐DN membrane indicate possible applicability towards biomedical applications, such as implantable power supplies that harvest energy from biological fluids. Previous reports have demonstrated similar RED systems in applications such as transdermal drug delivery [86] or biosensors [87]. For instance, an implantable RED device could be utilized to harvest energy in kidneys, where physiological salt concentration gradients are found in the renal artery and vein streams by virtue of kidney function to remove salt in maintaining osmolarity. Although physiological salinity gradients are low (e.g., 0.15/0.12 M NaCl), we found that the SRAP‐DN RED device could nonetheless generate a respectable power output of *ρ*_max_ = 0.18 Wm^−2^ in these conditions (Figure 3i), much higher than preceding reports of RED systems under the same conditions [88].

#### 2.4.3 Centimeter‐Scale RED Cell Arrays

Practical applications of RED cells will require them to be connected in parallel and/or series to amplify the voltage and current output. Toward this end, we developed 3D‐printed centimeter‐scale RED cells (Figure 4a–c) to facilitate evaluations of linking multiple SRAP‐DN RED cells together in the natural seawater/river water gradient. When two cells are connected in parallel, the current rises to 16.1 μA, double that of each individual cells having *I*_SC_ of ≈8 μA as expected (Figure 4a). Likewise, the output voltage of each cell (160 mV) sums to 320 mV when two cells are connected in series (Figure 4b). These results validate the potential to link together a sufficient number of these small‐scale RED cells in order to obtain a desired voltage and current output. For example, Figure 4c demonstrates a device consisting of 24 centimeter‐scale cells connected in series to obtain a high output voltage of 2.32 V, of practical use for powering small electronic devices.

#### 2.4.4 Gel‐Based Electrolytes for Flexible RED Devices

While the centimeter‐scale cells provide a proof of concept for miniaturization and power amplification, we envisioned that a gel‐based flexible thin‐film device may have even more practical value for applications such as wearable or implantable electronics. Therefore, we prototyped a simple flexible RED device by placing the SRAP‐DN membrane between two gelatin‐based films derived from seawater and river water at pH 12 (Figure 4d) to avoid liquid electrolytes. The three‐layer stack of hydrogels was encapsulated with copper tape and PET to fabricate a flexible thin‐film RED device, which exhibits a good energy‐harvesting performance (*ρ*_max_ = 9.1 Wm^−2^ and up to 245 Am^−2^ of current density, Figure 4e). Though favorable, the power output is lower than in the case of the alkaline liquid electrolyte, probably due to lower ion diffusion rates in gel state. The power and voltage remained stable at ∼140 mV and ∼8 Wm^−2^, respectively, over a 12 h period of continuous operation (Figure 4f), showcasing a high stability of the thin‐film device for prolonged use. We note that the Cu tape electrodes employed in this proof‐of‐concept device are not suitable for sustained operation, since Cu undergoes oxidative dissolution (Cu → Cu^2+^) at the anode, leading to electrode degradation and gel contamination, while the Cu/Cu^2+^ redox potential introduces a galvanic contribution to the measured open‐circuit voltage that is absent in the Ag/AgCl‐based measurements of Figures 2 and 3. For practical devices, electrochemically stable alternatives such as carbon‐based or Ag/AgCl electrodes would be required. The Cu and Ag/AgCl electrode systems differ qualitatively, not merely by a fixed redox‐potential offset. The Ag/AgCl electrodes used in Figures 2 and 3 are reversible to chloride and contribute a Nernstian electromotive force (*V*_elec_ ≈ 92 mV across the 50‐fold KCl gradient; see Supporting Information Section S2), whereas the Cu electrodes operating at pH 12 are not reversible to chloride and would in the ideal reversible limit contribute *V*_elec_ ≈ 0 mV from the salinity gradient, because the dominant Cu/Cu(OH)_2_ couple responds to [OH^−^] which is symmetric across the cell. Consistent with this expectation, the Cu‐based gelatin device yields *V*_oc_ ≈ 140 mV (Figure 4f), in the range of the membrane EMF inferred independently from the Ag/AgCl measurements at pH 12 after subtracting the Ag/AgCl electrode contribution. For these reasons, Figures 2 and 3 (Ag/AgCl in well‐defined KCl electrolytes) represent the authoritative source for intrinsic SRAP‐DN membrane performance, while Figure 4 (Cu in gelatin‐based electrolytes) is a form‐factor demonstration of mechanical flexibility and miniaturization.

#### 2.4.5 A Gel‐Based Flexible Thin‐Film Power Supply

As a culmination of the above investigations to optimize the SRAP‐DN hydrogel membrane for RED‐based blue energy harvesting and prototyping miniaturized and flexible devices derived thereof, we fabricated (Figure 4g) an encapsulated 24‐cell gel‐based power supply as a proof of concept for wearable or implantable applications of the technology. As shown in Figure 4h–i, the device is thin and flexible and continues to power a digital clock when it is deformed by bending with the hand. The 24 gelatin‐based cells are connected in series to exert an open circuit voltage of ∼2.2 V, which maintains stable power output for at least 5 min (Figure 4j). This proof of concept builds confidence for the prospect of low‐cost, low‐impact, water‐based materials for blue energy harvesting.

## 3 Conclusion

In summary, we created a tough slide‐ring asymmetric polyelectrolyte double network (SRAP‐DN) hydrogel membrane that possesses a gradient of space charge afforded by the unilateral polymerization of a highly entangled acrylic polyelectrolyte network into a tough slide‐ring hydrogel, which leads to a unique combination of high toughness, water content, and cation‐selective ionic transport properties. The SRAP‐DN hydrogel membrane exhibits a high *ρ*_max_ of 10.1 Wm^−2^ in the standard 50‐fold KCl gradient, well exceeding more conventional hydrogels and the commercialization benchmark for RED devices. The SRAP‐DN membrane also performs very well in a natural seawater/river water gradient and remains stable beyond 24 h in these conditions, indicating good stability, toughness, and biofouling resistance. The energy output can be enhanced dramatically to 12.8 Wm^−2^ in alkaline conditions (pH 12) or in more hypersaline conditions, even surpassing 20 Wm^−2^ in a 200‐fold KCl gradient. Conversely, the SRAP‐DN RED systems function well in low salinity gradients such as the 0.15/0.12 M NaCl gradient of high relevance to physiological applications. In addition, we demonstrated that the liquid‐electrolyte RED cells can be miniaturized to the centimeter scale, while millimeter‐scale thickness thin‐film devices can be prepared when the electrolyte is gelled with gelatin. As expected, connecting multiple devices in series or parallel enables amplification of the voltage or current, which enabled us to demonstrate preliminary power supply comprising 24 micro‐cells that produce well over 2 V of potential. Since this work lays a foundation for highly water‐rich hydrogel‐based energy‐harvesting devices that are cost‐effective, scalable, and eco/bio‐friendly, the SRAP‐DN membrane may be of interest for other clean energy devices such as moist‐electric generators (MEGs) [89, 90]. These promising results call for future research on several fronts to support potential translation to market and expansion of the application scenarios for these hydrogel membranes, including biofouling, bio‐compatibility, and bio‐degradation studies, evaluation of electrochemically stable electrode systems for sustained operation of the flexible device form factor, and direct characterization of how the membrane’s power density scales with effective test area across the micro‐ to macro‐aperture regimes.

## 4 Experimental Methods

### 4.1 Materials

Polyethylene glycol (PEG, MW 35 kDa, 818892), 2‐(hydroxypropyl)‐*α*‐cyclodextrin (HP‐*α*CD, 390690), acrylamide (AAm, A8887), 2‐acrylamido‐2‐methylpropanesulfonic acid (AMPS, 282731), 2‐hydroxy‐4‐(2‐hydroxyethoxy)‐2‐methyl‐propiophenone (Irgacure 2959, 410896), *N*,*N*’‐methylenebisacrylamide (MBA, M7279), dialysis membrane (MWCO 14 kDa, D9777), and gelatin from bovine skin Type B were purchased from Sigma Aldrich. Ethylenediamine anhydrous (98%, E0037), 3‐hydroxy‐1‐adamantane carboxylic acid (AdOH‐COOH, 96%, H1255), divinyl sulfone (DVS, 96%, D0959), and Rhodamine B dye (R0040, 95%) were purchased from TCI America. 4‐(4,6‐Dimethoxy‐1,3,5‐triazin‐2‐yl)‐4‐methylmorpholinium chloride (DMTMM, 99.1%) was purchased from Chem Impex International, Inc. *N*,*N*’‐Carbonyldiimidazole (CDI) was purchased from Oakwood Chemicals. Tetrahydrofuran (THF), phosphate‐buffered saline (PBS, pH 7.4), potassium chloride (KCl, 99%), sodium chloride (NaCl, 99%) hydrochloric acid (HCl, 12 N), and sodium hydroxide (NaOH) were purchased from Fisher Scientific. Dimethyl sulfoxide‐D6 (DMSO‐d6, 99.9%) was purchased from Cambridge Isotope Laboratories, Inc. Ethanol was purchased from Decon Laboratories, Inc. All materials were used as‐received without further purification. Amine‐terminated PEG (PEG(NH_2_)_2_) was synthesized according to a reported procedure [91]. HPPR was synthesized according to a reported one‐pot protocol [92] from PEG(NH_2_)_2_ (detail in Supplementary Information). Water was collected from MiliQ water purifier and degassed, purged with nitrogen prior to use. Natural sea water and river water were collected from the Pacific Ocean (Los Angeles, CA) and Boulder Creek (Boulder, CO) respectively and filtered through filter paper (Whatman Grade 1) prior to use. Artificial sea water was prepared following ASTM D1141‐98 standard. Ag/AgCl wire (*ϕ* 0.37 mm) was purchased from Warner Instruments (Harvard Bioscience Inc., MA). Silicone rubber sheet (with varying thickness 0.1–0.5 mm), PET adhesive tape (thickness 0.1 mm), PLA filaments, resistors (1‐10^6^ Ω), LED light, acrylic sheets, and Teflon sheets were purchased from Amazon.com Inc. Threaded rod (dia. 4 mm), wing nuts, copper tape, and alligator clips were purchased from local hardware shop (McGuckin Hardware, Boulder, CO).

#### 4.1.1 Synthesis of HPPR Single Network Hydrogel (HPPR‐SN)

A typical HPPR gel (with [HPPR] 38 wt% and [DVS] 2.5 wt%) was synthesized by dissolving HPPR polymer (150 mg) into 250 μL 0.05 M NaOH solution and divinyl sulfone (DVS) (10 mg). The solution was transferred into a mold made of Teflon sheet coated glass slides and silicon rubber spacer fixed with binder clips and maintained at 27°C for 48 h to allow gelation. Similarly, different HPPR gel formulations were synthesized by varying HPPR polymer and crosslinker concentration. Finally, the mold containing HPPR gels was stored in a sealed box till further use. Different HPPR‐SN hydrogel formulations were prepared varying HPPR polymer (18–38 wt%) and DVS crosslinker (1.5–4 wt%) concentrations.

#### 4.1.2 Synthesis of PAAm‐AMPS Single Network Hydrogel (PAAm‐AMPS‐SN)

PAAm‐AMPS‐SN hydrogel was synthesized by UV initiated photopolymerization of a pre‐gel solution. For a typical formulation of PAAM‐AMPS‐SN, 431 mg of AAm and 75 mg of AMPS (a [total monomer] of 26 M) was dissolved in degassed water (233μL) by vigorous mixing at 27°C. MBA crosslinker (0.001 mol% with respect to the [total monomer]) and Irgacure 2959 initiator (0.0005 mol% with respect to the [total monomer]) solutions were added to the above monomer solution. The solution was then purged with nitrogen and then sonicated at 30°C for 3 min. The pre‐gel solution was poured into a mold made up of two glass slides with a black covering on one side and a silicone rubber spacer fixed by binder clips. The PAAm‐AMPS‐SN hydrogel was formed by photopolymerization under UV light (*λ* = 365 nm, 5 mW cm^−2^) for 1 h. The hydrogel was peeled off from the mold and immersed into excess water for 24 h for equilibrium swelling before further tests. Similarly, different PAAm‐AMPS‐SN hydrogel samples were synthesized by varying total monomer concentration (15–26 M), MBA (0.5–2.0 × 10^−3^ mol%), and Irgacur 2959 initiator (0.25–1.0 × 10^−3^ mol%) concentrations in the pre‐gel solutions.

#### 4.1.3 Synthesis of SRAP‐DN Hydrogel

To synthesize SRAP‐DN, a pre‐dried HPPR‐SN hydrogel was soaked into the pre‐gel solution used to synthesize PAAm‐AMPS‐SN and then photopolymerized under UV light. For a typical formulation, a pre‐gel soaking solution was prepared by mixing 431 mg of AAm, 75 mg of AMPS, MBA crosslinker (0.001 mol% with respect to the [total monomer]) and Irgacure 2959 initiator (0.0005 mol% with respect to the [total monomer]) in degassed water (233 μL) by vigorous mixing at 27°C. Subsequently, a pre‐dried piece of HPPR‐SN gel was immersed into the pre‐gel solution and the container was kept in dark for 3 h at 27°C for swelling. Afterward, the swelled HPPR‐SN gel was placed between two glass slides with a black covering on one side and silicone rubber spacer, tightly clamped by binder clips and kept under UV irradiation (365 nm, 5 mW cm^−2^) for 1 h to form PAAm‐AMPS network into the HPPR network. Finally the sample was kept in water for 24 h till complete swelling before the tests. Similarly different SRAP‐DN hydrogels were synthesized by varying monomer ratios and MBA crosslinker in the pre‐gel soaking solutions.

#### 4.1.4 Mechanical Testing

For tensile test, a rectangular sample (20 × 5 × 0.25 mm^3^) was mounted to the solid rectangular fixtures (SRF) of a dynamic mechanical analyzer (MCR 702). The sample was stretched at a speed of 100 mm min^−1^ until failure. Stress (*σ*) value was obtained by dividing force by the initial cross‐sectional area of the sample. Strain (*λ*) value was calculated by dividing elongation by initial length of the sample. The stiffness (*E*) was calculated from the initial slope of the stress–stretch curve. Toughness (*U*) was calculated from the area under the tensile stress versus strain curve using Origin Pro software. To calculate fracture energy (*Γ*), a tensile stress versus strain data was recorded for a sample with a notch. Then, the area under the curve (*U*) was calculated until critical strain from where the crack grows catastrophically, and finally *Γ* was calculated by *Γ* = *H* × *U*, where *H* is the initial sample length. All of the tensile data was recorded inside a custom‐made humidity chamber to mitigate effects of drying. The data was reported by averaging five measurements and statistical analysis in Origin Pro software.

#### 4.1.5 Fabrication of RED Setup

Reverse electrodialysis (RED) measurements were conducted using a custom‐made assembly of two 3D‐printed compartments setup (detail in Supplementary Information). These compartments were filled with different concentrations of electrolyte solutions. The SRAP‐DN hydrogel membrane was sandwiched between two PET sheets having circular hole with a diameter of 200 μm. Effective test area (*S*) was controlled by adjusting the hole size in PET sheet. The *S* was approximately 3 × 10^4^ μm^2^. This sandwiched assembly was inserted between the two 3D printed compartments along with silicon rubber gaskets and compressed with acrylic sheets and screw‐nut assembly from both ends. Two custom‐made Ag/AgCl electrodes (area of 3.2 cm^2^) were inserted into the compartments and connected to the electrochemical workstation for further electrochemical studies.

#### 4.1.6 Fabrication of Flexible RED Power Supply

The RED based wearable device was fabricated by sandwiching SRAP‐DN hydrogel membrane between two gelatin‐based electrolytes with salinity gradient. Gelatin was dissolved (0.1 g/mL) in sea water and river water and pH was adjusted to 12 by adding NaOH. The solution was vigorously stirred at 70°C for 2 h and immediately drop cast on a polyethylene terephthalate (PET) tape along with copper tape electrodes and kept at room temperature for 5 min for gelation. Subsequently, the gelatin‐electrode layers with sea water and river water were encapsulated in a PET pouch along with the SRAP‐DN hydrogel membrane to fabricate a single cell. Similarly, a combination of 24 individual gelatin/SRAP‐DN based RED cells were connected in a series to fabricate one flexible and portable RED device. The gelatin‐sea water or river water pre‐gel was drop cast on a die‐cut silicone rubber mold having 24 wells attached with copper tape electrodes. 24 small pieces of the SRAP‐DN were inserted in between two PET sheets having micro‐holes. Then, all these layers were adhered by compressing and encapsulating in a PET pouch. The two copper tape terminals were attached to connectors for applications.

#### 4.1.7 Electrochemical Measurements

Electrochemical measurements of RED setup were conducted by BASi Epsilon EC electrochemical workstation. For conductance measurements, the two compartments were filled with aqueous KCl solutions (10^−6^–1 M), and linear sweep voltammetry (LSV) was performed to determine open circuit voltage (*V*_OC_) and short circuit current (*I*_SC_). Then, resistance (*R*) and conductance were calculated from *V*_OC_ and *I*_SC_ using Ohm’s law. Rectification ratio was calculated from the LSV plot (−1000 to 1000 mV) when two compartments were filled with 0.5 M KCl solutions. To determine the orientation of the SRAP‐DN hydrogel membrane, i.e., HD side or LD side facing high salinity, the two compartments were filled with 0.5 M and 0.01 M KCl aqueous solutions respectively and LSV was performed for a range of −250 to 250 mV with a scan rate of 0.005 V/s to investigate trans‐membrane current or *I*_SC_ in forward (HD side facing 0.5 M solution) and backward (HD side facing 0.01 M solution) orientation of the membrane. For power density (*ρ*) measurements, an external variable load resistor (*R*_L_) was connected into the circuit in series, and current (*I*) harvested in the system was measured simultaneously with varying *R*_L_. *ρ* was calculated from *I* ^2^ *R*_L_/*S*, where *S* denotes the effective testing area of the membrane. Current density (*j*) was calculated from *I*/*S*. Similarly, electrochemical measurements were conducted with different electrolyte conditions, natural sea water and river water, and with different SRAP‐DN hydrogels.

## Funding

This study was supported by the National Science Foundation (2106158 and 2023179).

## Conflicts of Interest

The authors declare no conflicts of interest.

## Supporting information

Supplementary Material

## Data Availability

The data that support the findings of this study are available from the corresponding author upon reasonable request.
