# Supplementary material for: Asymmetric Doping of a Polyelectrolyte Network Into a Tough Slide‐Ring Hydrogel Membrane to Enhance Sustainable Osmotic Energy Harvesting
Source: Small Sci. 2026 Jul 9;6(7):e70334. doi: 10.1002/smsc.70334 (PMC13353304; doi:10.1002/smsc.70334)
Supplement: Supplementary file 1 — Supplementary Material [file SMSC-6-e70334-s001.pdf]

Supporting Information

Asymmetric Doping of a Polyelectrolyte Network into a Tough Slide-Ring Hydrogel Membrane to Enhance Sustainable Osmotic Energy Harvesting

Subhankar Mandal[1], Ignacio Lorente Montero[1], Aseem Milind Visal[2], and Carson J. Bruns* [1,2,3]

[1]ATLAS Institute, University of Colorado Boulder, Boulder, CO, USA 80309
[2]Materials Science and Engineering Program, University of Colorado Boulder, Boulder, CO, USA 80309
[3]Paul M. Rady Department of Mechanical Engineering, University of Colorado Boulder, CO, USA 80309
*Corresponding Author Email Address: carson.bruns@colorado.edu

1 Experimental Section

Instrumentation

Mechanical properties were measured using a dynamic mechanical analyzer (MCR-702, Anton Paar). Electrical measurements were conducted in a electrochemical workstation, BASi Epsilon EC, Bioanalytical Systems Inc. Optical microscopy (Nikon Eclipse TE300) was used to image the effective hole diameter of the PET window and the hydrogel membrane. The charge distribution in the membrane was observed by fluorescence microscope (Nikon Eclipse TE300) equipped with Hg lamp and 530 nm green light filter cubes utilizing a dye tagged hydrogel membrane. Scanning Electron Microscope (Hitachi SU3500 VP) was utilized to image the morphology of the freeze dried SRAP-DN hydrogel membrane. Fourier transform infrared spectra (FTIR) was recorded on Thermo Scientific Nicolet iS20 with ATR accessories and used to analyze the chemical structure of the double network. Labconco 4.5 freeZone was used for lyophilization procedure. Nuclear magnetic resonance spectroscopy data was recorded with a Bruker Avance 300 MHz spectrometer and data processing was performed in MestreNova software. Makerbot Replicator 3D printer was used to fabricate the RED setup.

Synthesis of Amine-terminated PEG ($PEG(NH_2)_2$)

Amine-terminated Polyethylene glycol was synthesised following a previous report.[1] Polyethylene glycol (PEG, 10 g) was initially dried overnight at 50 °C under vacuum. Subsequently, the dried PEG was dissolved in dry tetrahydrofuran (THF) (40 mL) at 60 °C until homogenization was achieved. To this solution, N,N'-carbonyldiimidazole (CDI, 0.4 g) was added and left to react for 18 hours at 50 °C. Ethylene diamine (1.2 mL) was then introduced into the reaction mixture, and stirring was continued for 2 h at 50 °C. The resulting mixture was then treated with ethanol (40 mL) and cooled to 5 °C for precipitation of white solids. The precipitate was washed with cold ethanol during vacuum filtration. To further purify the product, the precipitate was dissolved in ethanol (80 mL) at 60 °C, re-precipitated at 5 °C, and washed with cold ethanol. This process was repeated once more. Finally, the precipitate was dried at 40 °C under vacuum for 12 h, yielding $PEG(NH_2)_2$.

One-pot synthesis of HPPR polymer

HPPR was synthesized according to a reported one-pot protocol.[2] A solution of hydroxypropyl α-cyclodextrin (HP-αCD) (1.416 g, 0.12 mol/L) was prepared by dissolving it in phosphate-buffered saline (PBS, 10 mL) solution. Separately, $PEG(NH_2)_2$ (1.65 g, 4.7 mmol/L) was dissolved in PBS (10 mL). These two solutions were then mixed and incubated at 4 °C for 2.5 days. Subsequently, the mixture was brought to room temperature and 30 mg of Adam-COOH and DMTMM (66 mg) was added, followed by stirring for 1 day at room temperature. An additional DMTMM (66 mg) was added, and the mixture was stirred for another day at room temperature. The solution was then dialyzed with water for 2 days, followed by lyophilization for 3 days to obtain the final product, denoted as HPPR polymer.

Swelling measurements

The swelling degree (S_W) was calculated using the following equation 1;

$$S_W = \frac{W_2}{W_1}(g/g) \tag{1}$$

where W_1 and W_2 are initial dry weight and final weights of hydrogel after swelling respectively. The samples were blotted well with tissue paper well before each measurement.

Water content (W) of the hydrogel was calculated by the following equation 2:

$$W = (m_s - m_d)/m_s \times 100\% \tag{2}$$

where, m_s is the weight of equilibrium swollen hydrogel and m_d is mass of the hydrogel after complete drying.

Morphological characterization

The freeze dried SRAP-DN hydrogel was cross-sectioned and observed under an optical microscope and under scanning electron microscope (SEM) to check the gradient morphology along the cross-section. The samples were sputter coated with a thin layer of platinum prior to SEM imaging. The asymmetric charge distribution and gradient along the SRAP-DN membrane was imaged by a dye-tagging experiment using a fluorescence active positively charged Rhodamine B dye. SRAP-DN hydrogel was immersed in a Rhodamine B solution for 1 h and then washed with water multiple times. Consequently, the cross-section was cut out and visualized under fluorescence microscope with 530 nm green light.

Quantitative analysis of electrode contributions to the open-circuit voltage

The Ag/AgCl electrodes used in our reverse-electrodialysis measurements are reversible to chloride ion, with half-cell potential $E = E^\circ - (RT/F)\ln(a_{\mathrm{Cl}^-})$. In a cell with unequal chloride activity in the two compartments, the two electrodes therefore contribute a Nernstian electromotive force,

$$V_{\mathrm{elec}} = \frac{RT}{F}\ln\left(\frac{a_{\mathrm{Cl}^-,\mathrm{high}}}{a_{\mathrm{Cl}^-,\mathrm{low}}}\right), \tag{3}$$

to the measured open-circuit voltage, where $a_{\mathrm{Cl}^-} = \gamma_\pm[\mathrm{Cl}^-]$ and $\gamma_\pm$ is the mean ionic activity coefficient. For the standard 50-fold KCl gradient (0.5 M / 0.01 M) at 298 K, using the Hamer–Wu activity coefficients $\gamma_\pm(0.5\,m) = 0.649$ and $\gamma_\pm(0.01\,m) = 0.902$,[3] we obtain $V_{\mathrm{elec}} \approx 92$ mV. The corresponding value computed without the activity-coefficient correction is ≈ 100 mV.

The measured cell open-circuit voltage decomposes as $V_{\mathrm{oc}} = V_{\mathrm{mem}} + V_{\mathrm{elec}}$, where the membrane EMF for a cation-selective membrane is

$$V_{\mathrm{mem}} = (2t_+ - 1)\,\frac{RT}{F}\ln\left(\frac{a_{\mathrm{high}}}{a_{\mathrm{low}}}\right), \tag{4}$$

and t_+ is the cation transport number in the membrane. The transport number is bounded by 0.5 (no selectivity) and 1.0 (ideal cation selectivity), and is the standard electrode-independent measure of intrinsic membrane selectivity.

Applying this decomposition to the V_{oc} values reported in Figure 2d gives $V_{\mathrm{mem}} \approx 8$ mV and $t_+ \approx 0.54$ for the forward orientation (HD side facing 0.5 M KCl), and $V_{\mathrm{mem}} \approx 93$ mV and $t_+ \approx 1.0$ for the reverse orientation (HD side facing 0.01 M KCl). The strong asymmetry in t_+ between forward and reverse orientations is a direct, electrode-independent signature of the diode-like ionic rectification reported in Figure 2c, which is itself measured with symmetric 0.5 M KCl and therefore free of any Nernstian electrode contribution.

The headline power density of 10.1 W m^{-2} reported for the 50-fold KCl gradient (Figure 2f) is measured in the forward orientation, where the membrane provides the lowest internal resistance (≈ 10 kΩ) and the Ag/AgCl electrodes contribute the majority of the cell EMF. Although in principle one can report a "membrane-only" power density by subtracting the electrode contribution from V_{oc}, we have followed the convention of the hydrogel-RED literature[4, 5, 6, 7, 8] and report device-level power densities for direct comparison with the values in Tables S1–S2, which are all measured using Ag/AgCl in chloride electrolytes. The chloride chemical potential difference is a genuine harvestable component of the salinity gradient free energy; the Ag/AgCl couple is the mechanism that converts it to electrical work, and removing it from the analysis would understate the actual energy harvested by the device.

For the gelatin-based flexible device of Figure 4 (Cu tape electrodes, pH 12), the relevant electrochemistry is qualitatively different from the Ag/AgCl case analyzed above. In alkaline electrolyte a $\mathrm{Cu(OH)_2/CuO}$ passivation layer forms on the Cu surface, and the dominant electrochemical couple is $\mathrm{Cu/Cu(OH)_2}$, with half-cell potential $E = E^\circ_{\mathrm{Cu/Cu(OH)_2}} - (RT/F)\ln(a_{\mathrm{OH}^-})$. Because both gelatin compartments are buffered to pH 12, the OH^- activity is symmetric across the cell and the ideal reversible Cu electrode contribution to the cell EMF from the salinity gradient is approximately zero; this is in contrast to the Ag/AgCl case where the chloride gradient generates the ~ 92 mV Nernstian contribution derived above. The measured V_{oc} of the Cu device (Figure **??**f, ≈ 140 mV) therefore primarily reflects the membrane EMF rather than an electrode contribution, and is consistent in magnitude with the membrane EMF inferred from the Ag/AgCl measurements at pH

12 after subtracting the electrode contribution. Cu additionally undergoes irreversible oxidative dissolution ($Cu \rightarrow Cu^{2+}$) at the anode under operation, contributing kinetically-controlled galvanic currents that complicate a strict thermodynamic interpretation; for this reason, Figure 4 is presented as a form-factor demonstration of flexibility and miniaturization rather than as a quantitative performance benchmark, with Figures 2–3 serving as the authoritative source for intrinsic SRAP-DN membrane performance.

Supplementary Figures

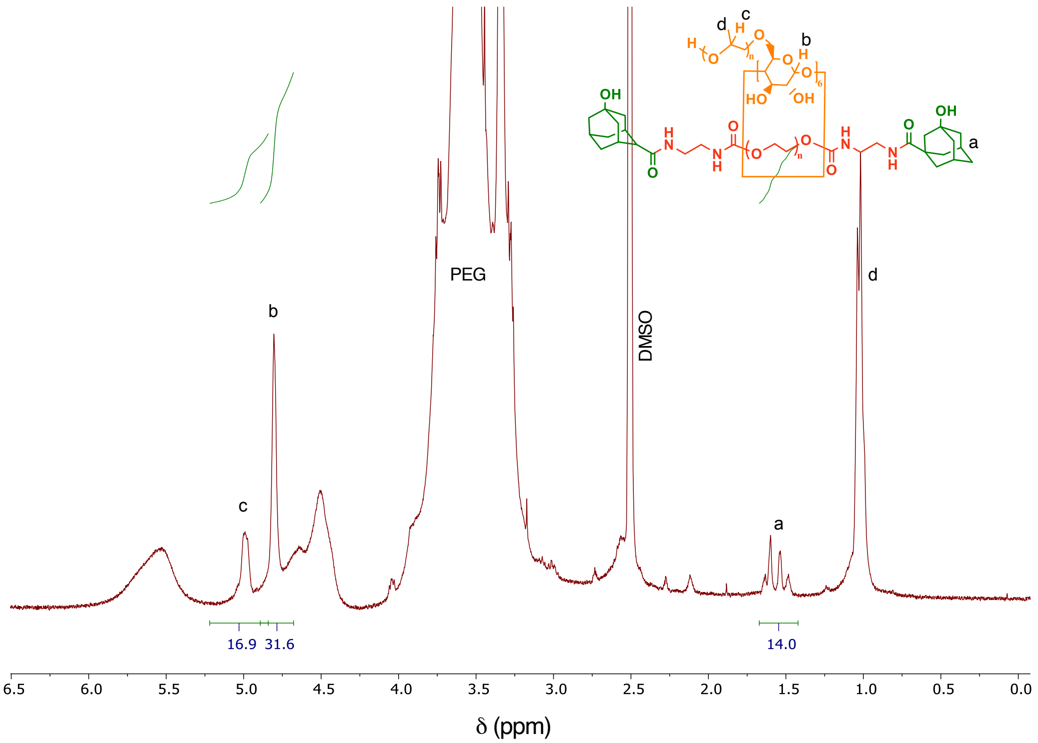

Figure S1: ^{1}H NMR of HPPR in $(CD_3)_2SO$ (294K, 300 MHz). Inclusion ratio calculated from the integration area (I) of C1-H (b) peak at δ4.8 ppm per PEG segment after normalization. Inclusion ratio was calculated to be 1.6%.

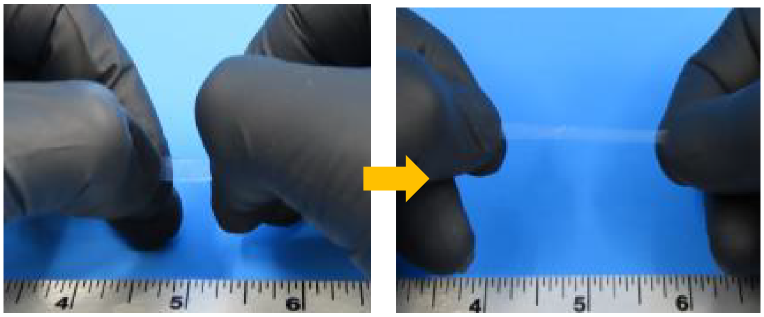

Figure S2: Digital images of SRAP-DN under tension, showing toughness and elasticity.

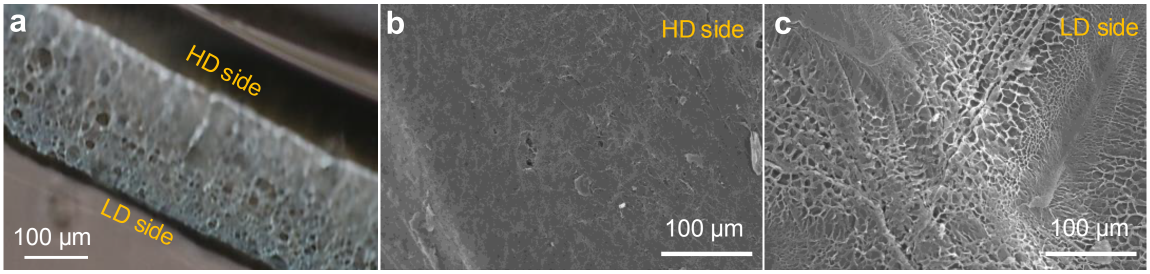

Figure S3: Micrographs of SRAP-DN hydrogel. a) Microscopic image of a cross-sectioned SRAP-DN hydrogel. SEM images of b) top surface or HD side and c) bottom surface or LD side of a SRAP-DN hydrogel membrane.

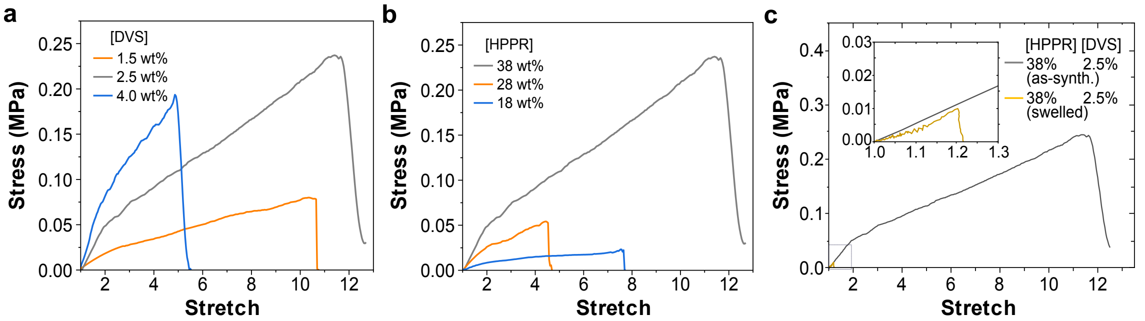

Figure S4: Mechanical properties of the single-network SRN hydrogel. Tensile traces of different HPPR-SN hydrogels a) varying [DVS] crosslinker at a fixed HPPR polymer content of 38 wt%, b) varying [HPPR] at a fixed [DVS] of 2.5 wt%. and c) Tensile stress versus strain plot of the as-synthesised and fully water swollen HPPR-SN with 38 wt% of HPPR and 2.5 wt% of DVS.

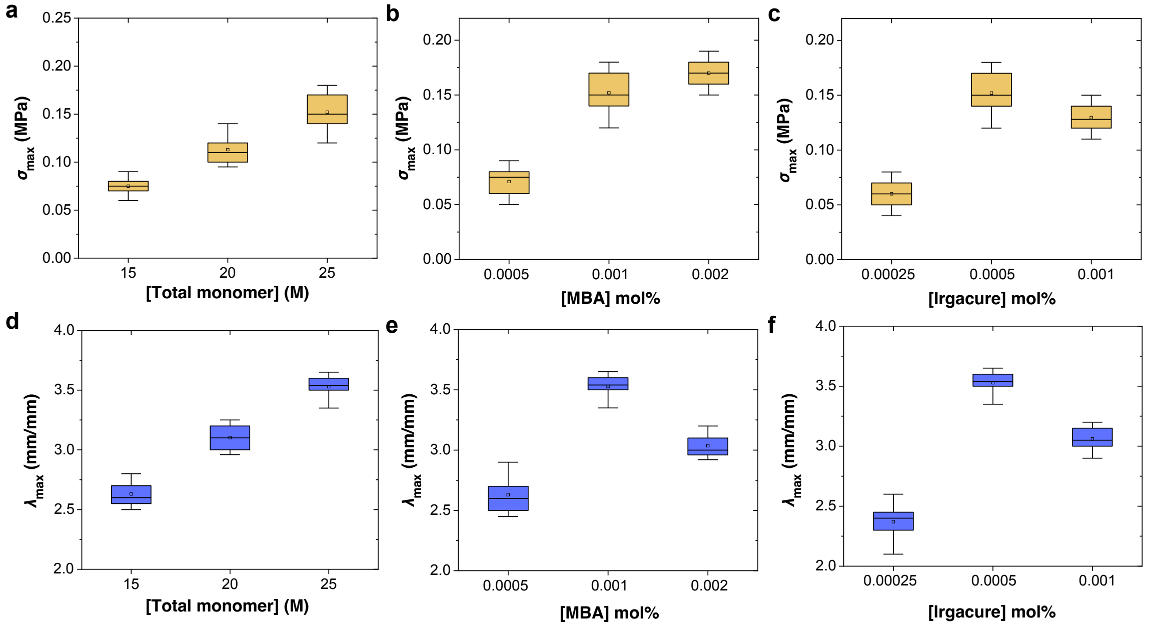

Figure S5: Mechanical properties of the single-network PAAm-AMPS hydrogel. a-b) Effect of total monomer concentration in the pre-gel solution on ultimate tensile stress and maximum stretchability of PAAm-AMPS-SN hydrogels at fixed [MBA] of 0.001 mol% and [Irgacure] 0.0005 mol%. c-d) Variation in ultimate tensile stress and maximum strain of PAAm-AMPS-SNs upon changing the [MBA] at fixed [monomer] of 25 M and [Irgacure] of 0.0005 mol%. e-f) Effect of [Irgacur] variation in the pre-gel solution on ultimate tensile stress and maximum strain of PAAm-AMPS-SN hydrogels at fixed [monomer] of 25 M and [MBA] of 0.001 mol%.

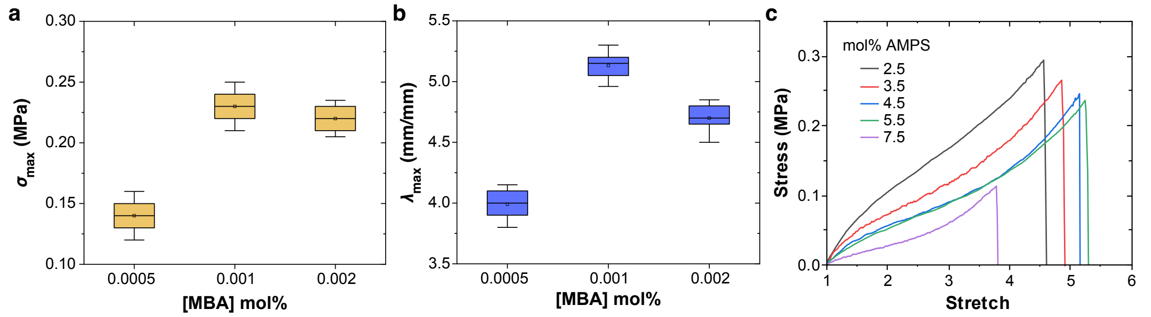

Figure S6: Mechanical properties of SRAP-DN. a-b) Variation in ultimate tensile stress and maximum strain of SRAP-DN while changing the [MBA] at fixed [monomer] of 25 M and [Irgacure] of 0.0005 mol% in the pre-gel soaking solution. c) Tensile traces of different SRAP-DN hydrogel samples with varying [AMPS] at fixed [total monomer] of 25 M, [MBA] of 0.001 mol% and [Irgacure] of 0.0005 mol%.

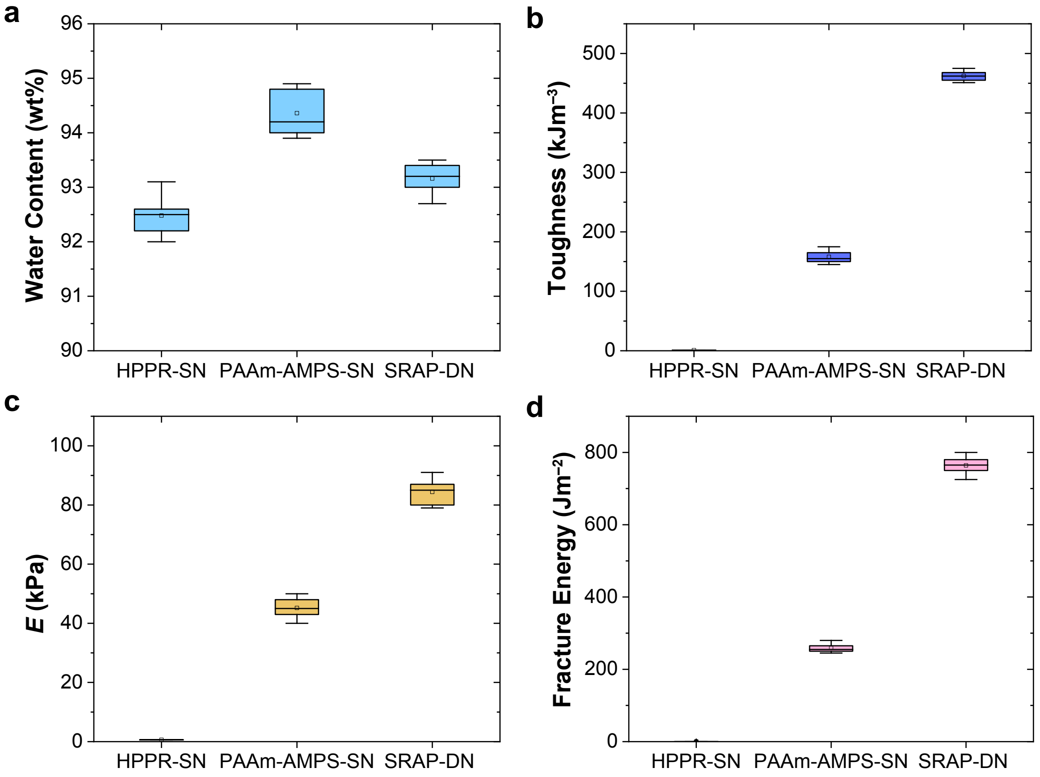

Figure S7: Comparison of single- and double-network membrane properties: a) Water content at equilibrium swelling state, b) Toughness, c) Stiffness and d) Fracture energy of SRN hydrogel, PAAm-AMPS hydrogel, and SRAP-DN hydrogel.

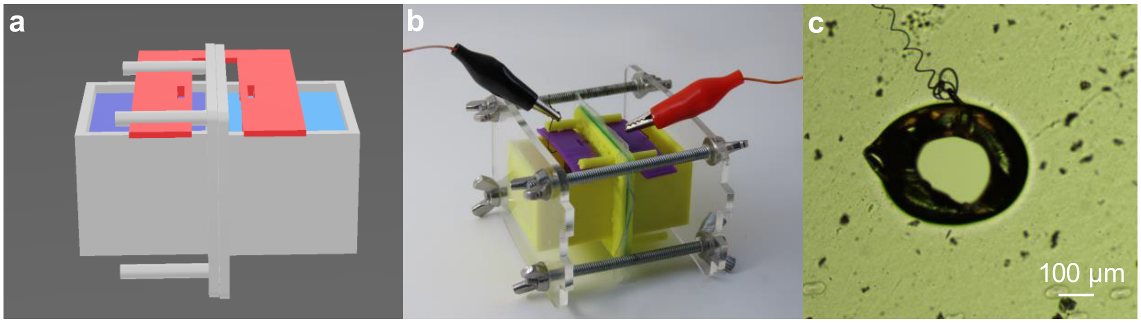

Figure S8: Fabrication of RED device. a) Rendered image of the RED setup while preparing for AutoCAD design and 3D printing. b) Digital image of the actual RED setup constituting 3D printed compartments, electrode holders, electrodes, membrane assembly and measurement terminals. c) Optical microscopic image of the PET sheet with the opening having effective effective area of 3x10-8 m2.

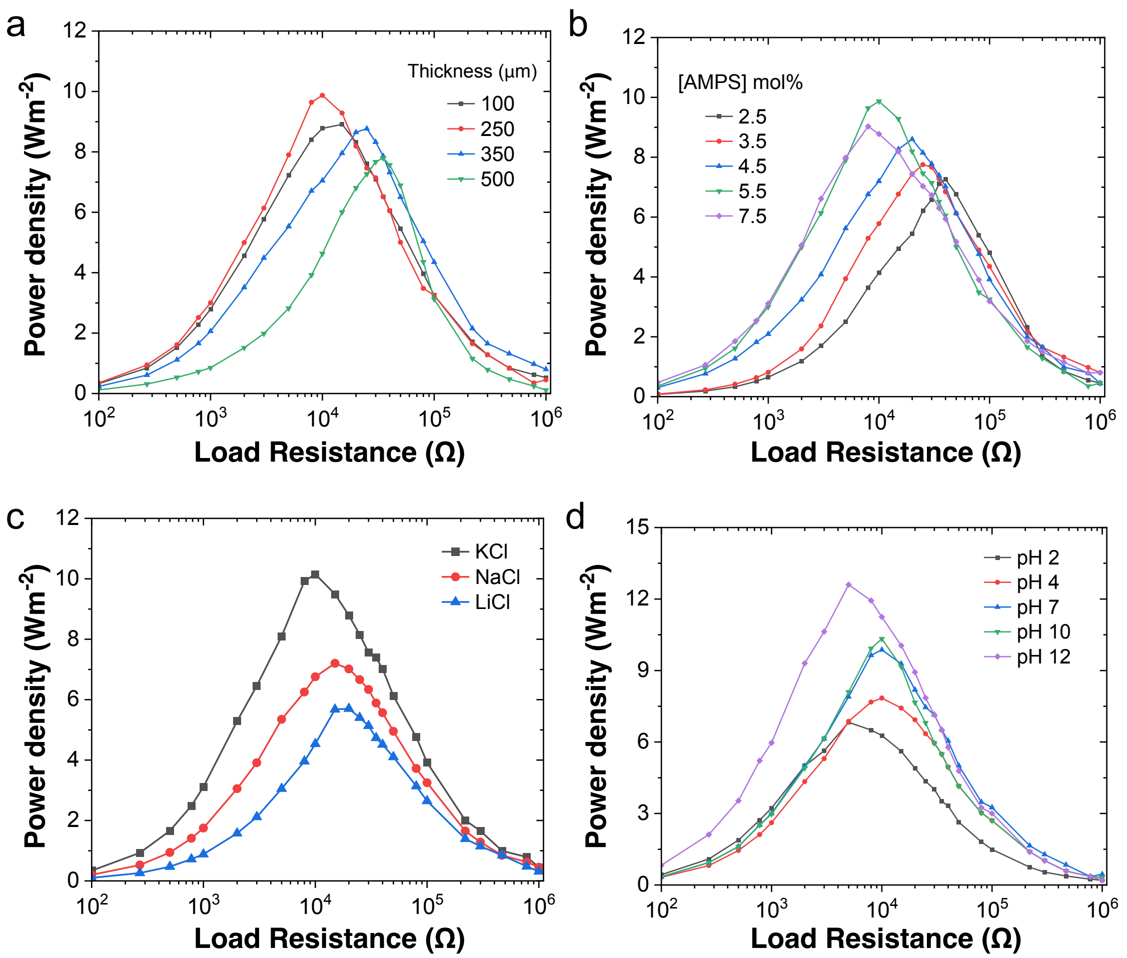

Figure S9: Plots of power density vs. load resistance for SRAP-DN based RED devices with varying parameters. a) Effect of membrane thickness on power density and load resistance in the standard 0.50 / 0.01 M KCl gradient. b) Effect of increasing the relative content of AMPS in the pre-gel soaking solution on the power density and load resistance in the standard 0.50 / 0.01 M KCl gradient with 250 μm-thick membranes. c) Effect of changing the cation from K^+ to Na^+ to Li^+ on the power density and load resistance in the standard 0.50 / 0.01 M salinity gradient with 250 μm-thick membranes. d) Effect of increasing alkalinity from pH 2 to pH 12 on the power density and load resistance in the standard 0.50 / 0.01 M KCl gradient with 250 μm-thick membranes.

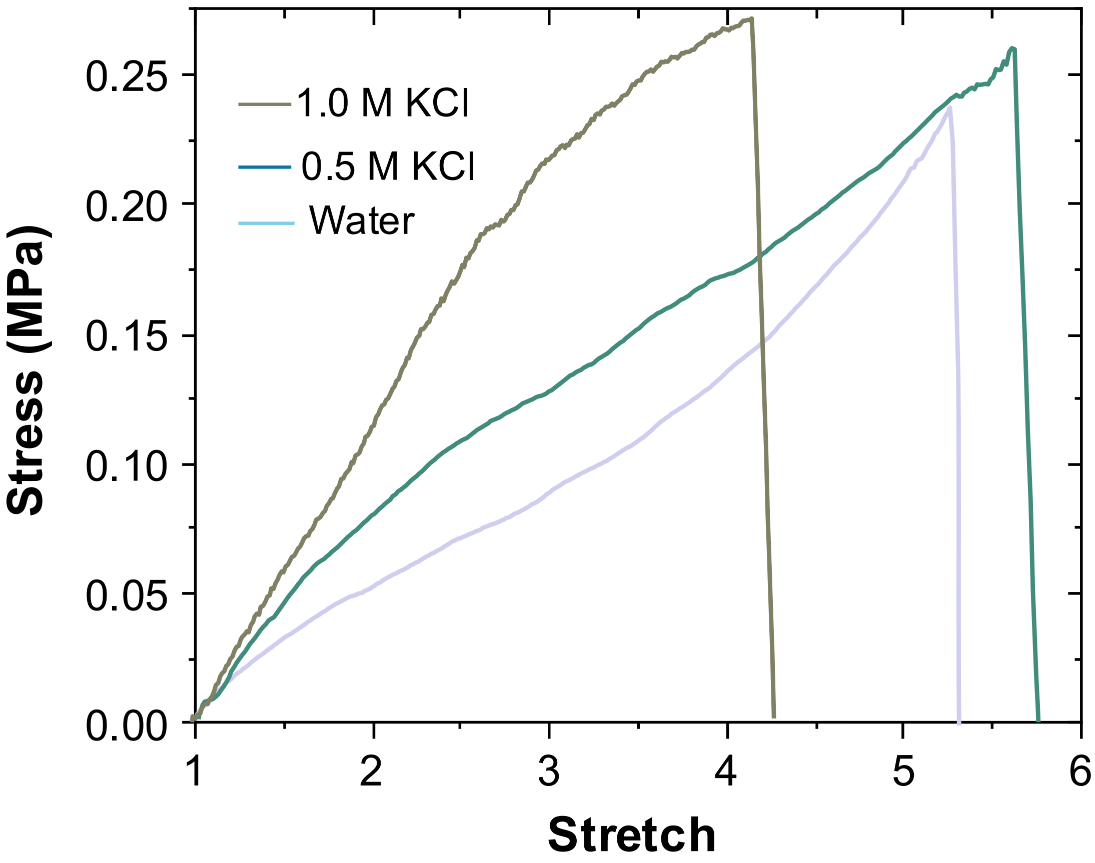

Figure S10: Stress-strain curves of SRAP-DN with increasing KCl salinity.

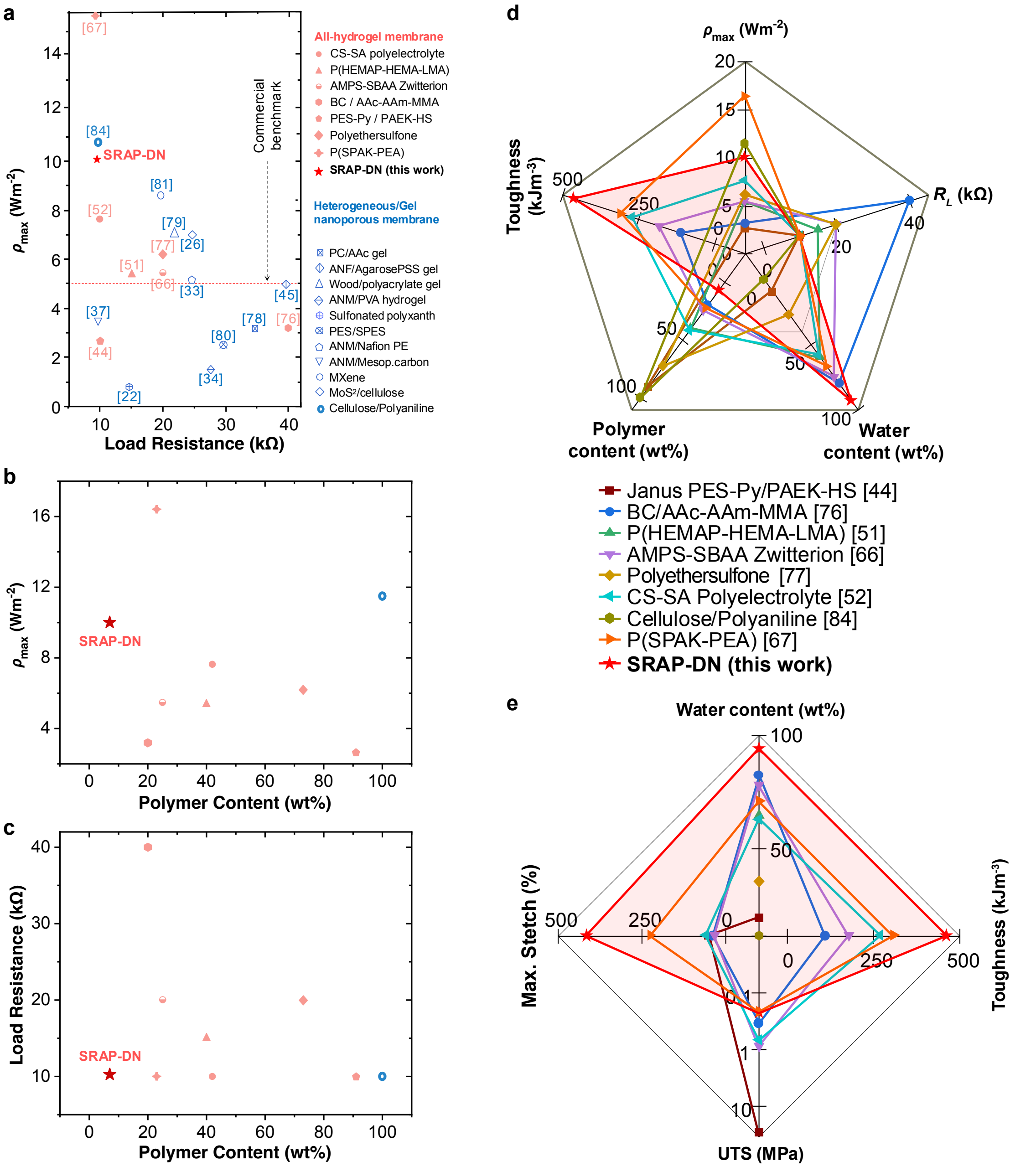

Figure S11: Comparison of SRAP-DN properties with other membranes reported in the literature.[22,26,33,34,37,44,45,50,51,66,77-82] (a) Scatter plot of maximum power density vs. load resistance for SRAP-DN and a number of hydrogel and non-hydrogel RED membranes. (b) Scatter plot of maximum power density vs. polymer content for all-hydrogel membranes. (c) Scatter plot of load resistance and maximum power vs. polymer concent for all-hydrogel membranes. (d) Spider plot comparing maximum power density, load resistance, water content, polymer content, and toughness for SRAP-DN and other hydrogel membranes. (e) Spider plot comparing water content, toughness, ultimate tensile stress (UTS), and maximum stretch among SRAP-DN and other reported hydrogel membranes.

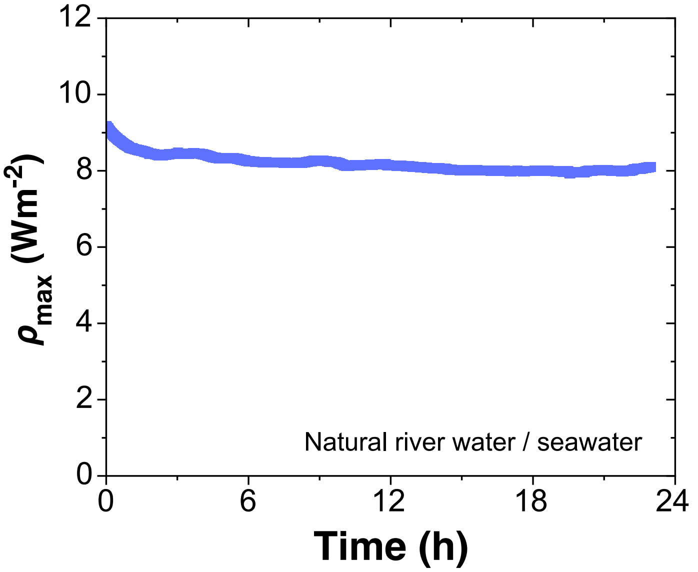

Figure S12: Stability of the SRAP-DN RED system over a 24-h period in the natural seawater / river water salinity gradient at 10 kΩ load resistance.

Table S1: Comparison of RED energy generating performance of different hydrogel and heterogeneous membranes operating in artificial sea and river water salinity gradient.

No	Material	ρ_{max} (Wm^{-2})	R_L ($k\Omega$)	Reference
1	CS-SA polyelectrolyte hydrogel	7.64	10	[4]
2	P(HEMAP-HEMA-LMA) hydrogel	5.38	15	[5]
3	AMPS-SBAA Zwitterionic hydrogel	5.44	20	[6]
4	BC/AAc-AAm-MMA hydrogel	3.18	40	[9]
5	Janus nanoporous PES-Py/PAEK-HS membrane	2.66	10	[10]
6	PES-SO_3H polyethersulfone	6.2	20	[11]
7	P(SPAK-PEA)	16.4	10	[7]
8	Cellulose/Polyaniline layered hydrogel	11.5	10	[8]
9	Polycarbonate membrane/ AAc-AAm-MMA hydrogel	3.18	35	[12]
10	Amaridnanofibrous membrane (ANF)/Agarose-PSS hydrogel	4.97	40	[13]
11	Wood-Sodiumpolyacrylate hydrogel membrane	7.01	22	[14]
12	Allumina nanochannel membrane (ANM)/PVA hydrgel	1.5	28	[15]
13	Sulphonated polyxanthene SPX membrane	0.8	15	[16]
14	Polyether sulfone PES/SPES	2.5	30	[17]
15	ANM/Nafion polyelectrolyte	5.13	25	[18]
16	ANM/Mesoporous carbon	3.5	10	[19]
17	MXene	8.6	20	[20]
18	MoS_2/Sulfonated cellulose nanofibre	7.05	25	[21]
19	Heterolayer-SMP-AA hydrogel/organogel	46.37	5	[22]
20	SRAP-DN hydrogel	10.1	10	This work

Table S2: Comparison of RED energy generating performance and polymer contents of different polymer network membranes against artificial sea and river water salinity gradient.

**Material**	ρ_{max} (Wm^{-2})	R_L $(k\Omega)$	**Water content (wt%)**	**Polymer content (wt%)**	**Reference**
Janus PES-Py/PAEK-HS	2.66	10	9	91	[10]
BC/AAc-AAm-MMA hydrogel	3.18	40	80	20	[9]
P(HEMAP-HEMA-LMA) hydrogel	5.38	15	60	40	[5]
AMPS-SBAA Zwitterionic hydrogel	5.44	20	75	25	[6]
PES-SO3H polyethersulfone	6.2	20	27	73	[11]
CS-SA polyelectrolyte hydrogel	7.64	10	58	42	[4]
SRAP-DN hydrogel	10.1	10	93	7	This work
Cellulose/Polyaniline layered network	11.5	10	0	100	[8]
P(SPAK-PEA) hydrogel	16.4	10	67	23	[7]

Table S3: Comparison of RED energy generating performance against artificial sea and river water salinity gradient and polymer contents along with its mechanical properties of different hydrogel network membranes.

**Material**	ρ_{max} (Wm^{-2})	R_L $(k\Omega)$	Water content (wt%)	Polymer content (wt%)	Mech. properties			Ref.
					UTS (MPa)	λ (%)	Toughness (kJm^{-3})	
Janus PES-Py/PAEK-HS	2.66	10	9	91	30	50	-	[10]
BC/AAc-AAm-MMA hydrogel	3.18	40	80	20	0.35	40	110	[9]
P(HEMAP-HEMA-LMA) hydrogel	5.38	15	60	40	-	-	-	[5]
AMPS-SBAA Zwitterionic hydrogel	5.44	20	75	25	0.9	33	180	[6]
PES-SO_3H polyethersulfone	6.2	20	27	73	-	-	-	[11]
CS-SA polyelectrolyte hydrogel	7.64	10	58	42	0.7	60	270	[4]
Cellulose/Polyaniline hydrogel	11.5	10	0	100	-	-	-	[8]
P(SPAK-PEA) hydrogel	16.4	10	67	23	0.22	225	308	[7]
BMAP hydrogel	5.99	27	50	50	-	-	-	[23]
**SRAP-DN hydrogel**	10.1	10	93	7	0.23	415	462	This work

References

[1] S. Zhou, J. Wang, G. Wang, Z. Jiang, H. Ren, *Eur. Polym. J.* **2017**, *90* 312.

[2] T. Noritomi, L. Jiang, H. Yokoyama, K. Mayumi, K. Ito, *RSC Adv.* **2022**, *12*, 7 3796.

[3] W. J. Hamer, Y.-C. Wu, *J. Phys. Chem. Ref. Data* **1972**, *1*, 4 1047.

[4] G. Bian, N. Pan, Z. Luan, X. Sui, W. Fan, Y. Xia, K. Sui, L. Jiang, *Angew. Chem. Int. Ed.* **2021**, *60* 20294.

[5] W. Chen, Q. Wang, J. Chen, Q. Zhang, X. Zhao, Y. Qian, C. Zhu, L. Yang, Y. Zhao, X.-Y. Kong, et al., *Nano Lett.* **2020**, *20*, 8 5705.

[6] K.-T. Huang, W.-H. Hung, Y.-C. Su, F.-C. Tang, L. D. Linh, C.-J. Huang, L.-H. Yeh, *Adv. Funct. Mater.* **2023**, *33*, 19 2211316.

[7] Y. Wang, H. Jiang, Y. Zhang, Z. Song, Z. Chen, K. Sui, W. Fan, P. Qi, *Chem. Eng. J.* **2024**, *481* 148512.

[8] Z. Xie, Z. Xiang, X. Fu, Z. Lin, C. Jiao, K. Zheng, M. Yang, X. Qin, D. Ye, *ACS Energy Lett.* **2024**, *9*, 5 2092.

[9] Z. Sun, Y. Kuang, M. Ahmad, Y. Huang, S. Yin, F. Seidi, S. Wang, *Carbohydr. Polym.* **2023**, *305* 120556.

[10] X. Zhu, J. Hao, B. Bao, Y. Zhou, H. Zhang, J. Pang, Z. Jiang, L. Jiang, *Sci. Adv.* **2018**, *4*, 10 eaau1665.

[11] Y. Sun, T. Dong, C. Lu, W. Xin, L. Yang, P. Liu, Y. Qian, Y. Zhao, X.-Y. Kong, L. Wen, et al., *Angew. Chem. Int. Ed.* **2020**, *59*, 40 17423.

[12] W. Chen, Q. Zhang, Y. Qian, W. Xin, D. Hao, X. Zhao, C. Zhu, X.-Y. Kong, B. Lu, L. Jiang, et al., *ACS Cent. Sci.* **2020**, *6*, 11 2097.

[13] Z. Zhang, L. He, C. Zhu, Y. Qian, L. Wen, L. Jiang, *Nat. Commun.* **2020**, *11*, 1 875.

[14] J. Chen, Y. Liu, D. Wang, H. Zhi, J. Tang, A. Shen, N. Li, Y. Zhang, H. Liu, G. Xue, *Nano Energy* **2022**, *104* 107981.

[15] H.-C. Yang, et al., *J. Mater. Chem. A* **2019**, *7*, 47 26791.

[16] Q. Zhu, Y. Li, Q. Qian, P. Zuo, M. D. Guiver, Z. Yang, T. Xu, *Energy Environ. Sci.* **2022**, *15*, 10 4148.

[17] X. Huang, Z. Zhang, X.-Y. Kong, Y. Sun, C. Zhu, P. Liu, J. Pang, L. Jiang, L. Wen, *Nano Energy* **2019**, *59* 354.

[18] C.-W. Chang, C.-W. Chu, Y.-S. Su, L.-H. Yeh, *J. Mater. Chem. A* **2022**, *10*, 6 2867.

[19] J. Gao, W. Guo, D. Feng, H. Wang, D. Zhao, L. Jiang, *J. Am. Chem. Soc.* **2014**, *136*, 35 12265.

[20] L. Ding, M. Zheng, D. Xiao, Z. Zhao, J. Xue, S. Zhang, J. Caro, H. Wang, *Angew. Chem. Int. Ed.* **2022**, *61*, 41 e202206152.

[21] K. Zou, H. Ling, Q. Wang, C. Zhu, Z. Zhang, D. Huang, K. Li, Y. Wu, W. Xin, X.-Y. Kong, L. Jiang, L. Wen, *Nat. Commun.* **2024**, *15*, 1 10231.

[22] W. Chen, K. Zhou, Z. Wu, L. Yang, Y. Xie, X. Meng, Z. Zhao, L. Wen, *J. Am. Chem. Soc.* **2024**, *146*, 19 13191.

[23] Z. Zhang, T. Zhou, X.-Y. Kong, Y. Wu, W. Xin, Y. Cui, L. Yang, T. Li, X. Li, Q. Wang, et al., *Nano Res.* **2023**, *16*, 8 11288.
